# Supplementary material for: tRNA lysidinylation is essential for the minimal translation system in the Plasmodium falciparum apicoplast
Source: EMBO Rep. 2025 Mar 20;26(9):2300–22. doi: 10.1038/s44319-025-00420-w (PMC12069591; doi:10.1038/s44319-025-00420-w)
Supplement: Supplementary file 1 — Appendix [file 44319_2025_420_MOESM1_ESM.pdf]

Appendix

tRNA lysidinylation is essential for the minimal translation system in the *Plasmodium falciparum* apicoplast

Rubayet Elahi[1,2,*], Sean T. Prigge[1,2,*]

[1] Department of Molecular Microbiology and Immunology, Johns Hopkins University, Baltimore, Maryland, USA
[2] The Johns Hopkins Malaria Research Institute, Baltimore, Maryland, USA

* Corresponding authors

Rubayet Elahi
Email: aelahi3@jhu.edu
Telephone: +1 443 287 6695
ORCID ID: 0000-0002-1561-5257

Sean T. Prigge
Email: sprigge2@jhu.edu
Telephone: +1 443 287 4822
ORCID ID: 0000-0001-9684-1733

Table of Contents

Appendix Figure S1. Multiple sequence alignment of TilS orthologs from *Plasmodium falciparum* (*Pf*TilS), *Synechocystis* sp. (*Sy*TilS), *Aquifex aeolicus* (*Aa*TilS), *Mycoplasma genitalium* (*Mg*TilS), *Escherichia coli* (*Ec*TilS), *Geobacillus kaustophilus* (*Gk*TilS), and *Arabidopsis thaliana* (*At*RSY3) 3

Appendix Figure S2. Pathogenic apicomplexans have TilS orthologs 5

Appendix Figure S3. The phylogenetic relationship among TilS orthologs 6

Appendix Figure S4. Uncropped image of immunoblot shown in Fig. 4D 7

Appendix Figure S5. Sequence alignment between *Archaeoglobus fulgidus* TiaS (*Af*TiaS) and *P. falciparum* TilS (*Pf*TilS) 8

Appendix Figure S6. Sequence of codon-modified full-length *pftils* (in blue font) as synthesized 9

Appendix Figure S7. Sequence of codon-modified N-terminally truncated *pftils* (in blue font) as synthesized 10

Appendix Table S1. Primers used in this study 11

Appendix Table S2. Proteins used for phylogenetic analysis presented in Appendix Fig. S3 14

Appendix Table S3. tRNAs used for phylogenetic analysis presented in Fig. EV2 16

```
PfTilS MYLLCIFLYILSFLCFTICIKRKQNVSKRWQFFIPHINKEGLYNIMDKKIIRVNKNKRFHRNVVCSNRLSEFIDRDININNKMKNIKELKKKHIDNNILIKADIFSNIIEKKNNSNLFFKKFCSFINMEY 130
SyTilS ---------------------------------------------------------------------------------------------------------------------------------- 0
AaTilS ---------------------------------------------------------------------------------------------------------------------------------- 0
MgTilS ---------------------------------------------------------------------------------------------------------------------------------- 0
EcTilS ---------------------------------------------------------------------------------------------------------------------------------- 0
GkTilS ---------------------------------------------------------------------------------------------------------------------------------- 0
AtRSY3 ---------------------------------------------------------------------------------------------------------------------------------- 0

PfTilS DETTKDLEELKINKRLKNNNNDNNNNNNNNMYDNKSNNIHNNNNNNNSDNLIKCIKKNQD---------------VLQLVEGYWLLTLKNKYMFSFLKKKKKIIFSVSSGVDSLCLLYSFIFVIYKILIS 244
SyTilS --------------------------------------------------------------------------MAWTLLHSKLHQNLQRR---FPELQGTRILVAVSGGQDSLCLLHLLNDLTKQWQ-- 51
AaTilS -------------------------------------------------------------------------MNPESRVIRKVLALQNDE---KIFSGERRVLIAFSGGVDSVVLTDVLLKLKNYFS-- 52
MgTilS -----------------------------------------------------------------------------------------------MASEKQYIAGVSGGSDSMLMLKLYQKKIA------ 29
EcTilS -------------------------------------------------------------------------------------MTLTLN---RQLLTSRQILVAFSGGLDSTVLLHQLVQWRTENP-- 40
GkTilS -------------------------------------------------------------------------------MIDKVRAFIHRH---QLLSEGAAVIVGVSGGPDSLALLHVFLSLRDEWK-- 46
AtRSY3 -----------MARGLSLCSN---GRKSSTLLSNSIPRVS--ISFSPKTLFTCSYPLQSRHPKFSQRLFCNHACVPETVDETRYKELFNKRMDMAGLKPHNRIALGVSGGPDSMALCVLTAKWKTEGL-- 112

PfTilS MIYKNKSYFGFMNKINSVYSFTHEDIIDIIKEYRYENNVSFFLSILSKIIVIYCHHNTRKE-CTSEMYFLKNICKKFGIHFKSKKLTEKSIQKLNVINLKTKKKNYESNVNKKININMMKNKKNFLLLAR 373
SyTilS ---------W------------------------------------HL-AVAHCDHRWPTDAGIAD--HVQGLAQ---------GY-------------------------------------------- 80
AaTilS ---------L------------------------------------KEVALAHFNHMLRES-AERDEEFCKEFAKERNMKIFVGKE-------------------------------------------- 92
MgTilS -------------------------------------------------CVVHVNYNTRST-SLRDQKLVEQYCQKLNIPLVVHTVDPDL---------------------------------------- 69
EcTilS ---------G------------------------------------VALRAIHVHHGLSAN-ADAWVTHCENVCQQWQVPLVVER--------------------------------------------- 79
GkTilS ---------L------------------------------------QV-IAAHVDHMFRGRESEEEMEFVKRFCVERRILCETAQI-------------------------------------------- 86
AtRSY3 ---------SCVNK---------------------------TDGFIDGLVAIVVDHGLRQESKDEAELVCSRVS-QMGIRCEIASC-------------------------------------------- 161

PfTilS TWRRNIYVHLSNDILKRDMNNNIYHNNKMKDSHNNNNNNDNNNTSMKDPLNMLDAYTYEKYIYDINNYIKITNKKCITNVLIKKESFSKEYSNDSIMRKKKKEGLLFNNIMNLQNNNIKIINNTCSNNIY 503
SyTilS ---------------------------------------------------------------KLP-------------------YFQR----------DAQDL------PQTEA------------AAR 100
AaTilS ---------------------------------------------------------------DVR-------------------AFAK----------EN--R------MSLEE------------AGR 110
MgTilS -------------------------------------------------------------------------------------VWKK----------------------NFQN------------QAR 80
EcTilS ---------------------------------------------------------------------------------------VQ----------LAQEG------LGIEA------------QAR 94
GkTilS ---------------------------------------------------------------DVP-------------------AFQR----------SA--G------LGAQE------------AAR 104
AtRSY3 ---------------------------------------------------------------D----------------------WVD----------GRPKL------GHLQE------------AAR 178

PfTilS NMKYTNVFLNKYCILKKKIKSIVFLGHHQNDNNETVLLQFFRGVFLKNLRGIKFLTYYK--------------NCLLYRPFIKLNKLHLYRYMQLINKTWNFDSSNNNMSISRNFIRNVVIPNITHMLKD 619
SyTilS HWRYH--AL--TAIAKAENFPVVMTGHTQSDRAETLLFNLVRGSGSDGLQAMNWVRNLEE-------SGSDKSPIRLIRPLLEISRQETGDFCQQQQLSVWEDVLNEKLTYRRNRIRGELIPYLKKHFNP 219
AaTilS FLRYK--FL--KEILESEGFDCIATAHHLNDLLETSLLFFTRGTGLDGLIGFLPKEE------------------VIRRPLYYVKRSEIEEYAKFKGLRWVEDETNYEVSIPRNRIRHRVIPELKR-INE 217
MgTilS KIRFDQ--F--KKTAKLYQTNKLLLAHHRDDFIEQAKMQLDAKKR-AVYYGIKTRCELY--------------GLKIYRPLMKYWKDEIIALCRQDHIPYEIDETNKLPIYKRNEVRLEIEKWSKI-EKE 190
EcTilS QARYQ--AF--ARTLLP--GEVLVTAQHLDDQCETFLLALKRGSGPAGLSAMAEVSEFA--------------GTRLIRPLLARTRGELVQWARQYDLRWIEDESNQDDSYDRNFLRLRVVPLLQQ-RWP 203
GkTilS ICRYR--FF--AELMEKHQAGYVAVGHHGDDQVETILMRLVRGSTSKGYAGIPVKRPFH--------------GGYLIRPFLAVSRAEIEAYCRQMGLSPRCDPSNEKDDYTRNRFRHHIVPLLRQ-ENP 215
AtRSY3 EMRYE--MI--SNVCFRQQIEVLLIAHHADDQAELFILRLSRSSGVLGLAGTAFASEIFSRNLQLDAKHMKNQSIRLVRPLLDLWKEDMYKICQWGRQDWVEDPTNRSQLFVRNRIRTSIGNLES----G 300

PfTilS KSYKNRENHNIDKEINEKNDKHMLDDNVCVDYHDETPLHNKKEMNKYDHVEISKNNIVNKNNELKNQHVYYVNTSLDRRLKNVLRQTTNLENYLNYYD---------NMFFTYLKKKYYKRCMSTTKKIT 739
SyTilS -QVEK----SLAQTVELLTAEVAYLEQVSGEIYQTLSQTDQ------KSLNCRL---------------LS------QK-PLALQRRIIRQFLQSCQ---------------------SQ---SPNF-E 290
AaTilS -NLED----TFLKMVKVLRAEREFLEEEAQKLYKEVK--------KGNCLDVKK---------------LK------EK-PLALQRRVIRKFIGEKD---------------------YEK-VEL----- 285
MgTilS QFY-----------IAICAMNKTIAQKLFVLMKK-----AKKWLLQPDVRELKRFSIIDQKQLIYSYLIYHKINV---------NGEKIDAILDFIQPSQQKQYRLQNDIFLMVK----NQCLALLYKS- 290
EcTilS -HFAE----ATARSAALCAEQESLLDELLADDLAHCQSPQ-------GTLQIVP---------------ML------AM-SDARRAAIIRRWLAG-Q---------------------NAP-MPSRDALV 276
GkTilS -RLHE----RFQQYSEMMAEDEQFLEELAADALNKVMEKQHR----DAALSIGP---------------FL------EL-PRPLQRRVLQLLLLR-L---------------------YGG-VPPTLTSV 291
AtRSY3 -SFKS----ELQAVISECRRTRSFVDKVCTDLIHQTVTVTDK---GYAILDLERLNPSGVKDICLSKYLFAVLQFISQR-QRPIRGNTSKLLLNYIR-----------------------A-IPCRTSLT 397
```

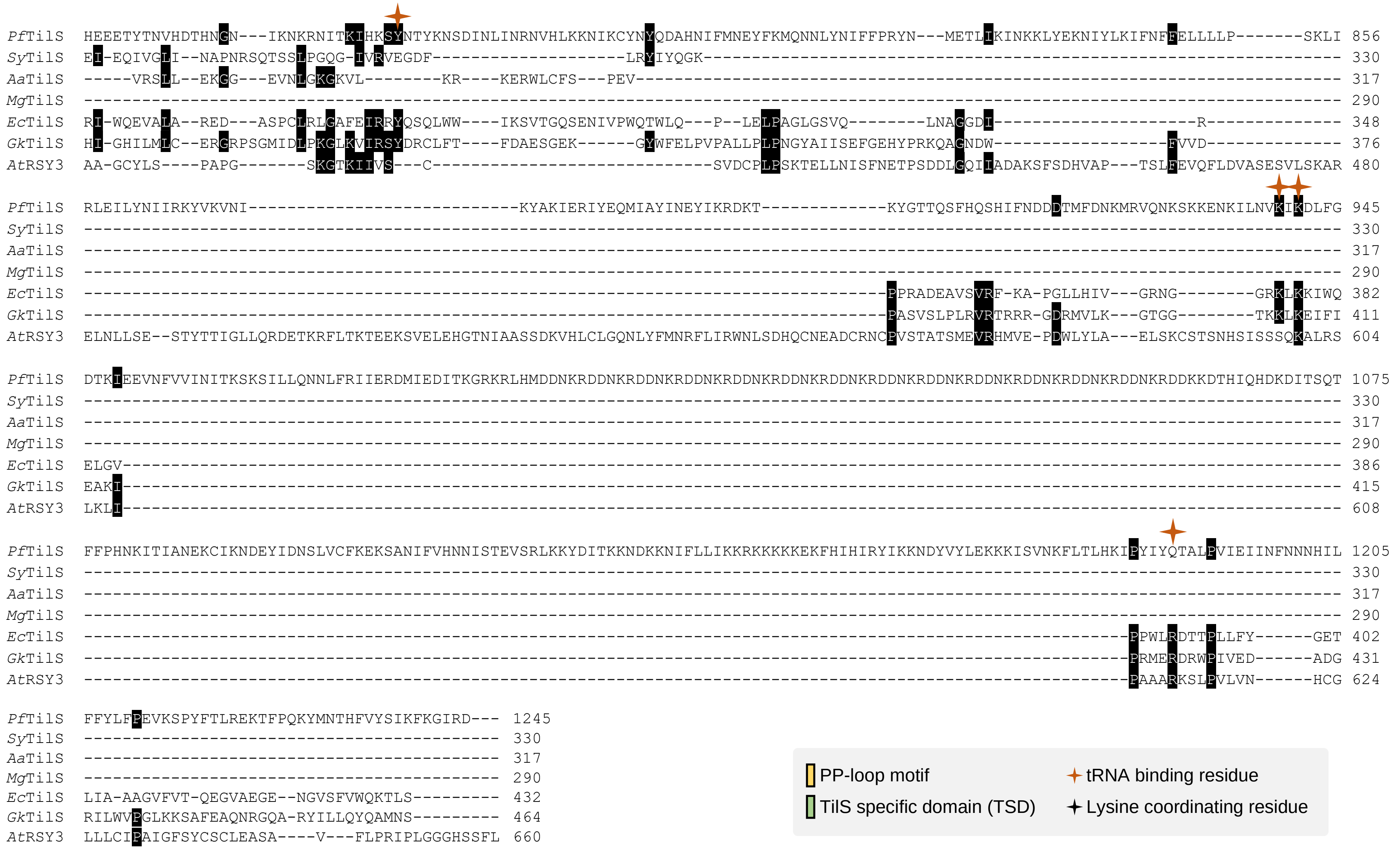

Appendix Figure S1. Multiple sequence alignment of TilS orthologs from *Plasmodium falciparum* (*Pf*TilS), *Synechocystis* sp. (*Sy*TilS), *Aquifex aeolicus* (*Aa*TilS), *Mycoplasma genitalium* (*Mg*TilS), *Escherichia coli* (*Ec*TilS), *Geobacillus kaustophilus* (*Gk*TilS), and *Arabidopsis thaliana* (*At*RSY3). Alignment output was visualized using Boxshade to highlight conserved residues. Amino acid residues and domains important for lysidinylation activity of TilS are depicted as in **Fig. 1E**.

Apicomplexans	Gene ID	Identity to *Pf*TilS	E-value	Experimental localization
Toxoplasma gondii	TGME49_215100	30%	1e-8	Apicoplast*
Eimeria tenella	ETH2_0718500	23%	0.025	N.D.
Neospora caninum	NCLIV_052110	30%	8e-10	N.D.
Babesia microti	BMR1_01G01110	39%	1e-11	N.D.
Theileria annulata	TA03600	30%	2e-4	N.D.

* Identified in the apicoplast fraction from *T. gondii* via hyperLOPIT (Barylyuk *et al.*, 2020); N.D., not determined

Appendix Figure S2. Pathogenic apicomplexans have TilS orthologs. TilS orthologs were identified in pathogenic apicomplexans using *P. falciparum* TilS as the query.

Reference:

Barylyuk K, Koreny L, Ke H, Butterworth S, Crook OM, Lassadi I, Gupta V, Tromer E, Mourier T, Stevens TJ et al (2020) A comprehensive subcellular atlas of the *Toxoplasma* proteome via hyperLOPIT provides spatial context for protein functions. *Cell Host Microbe* 28: 752-766.e759

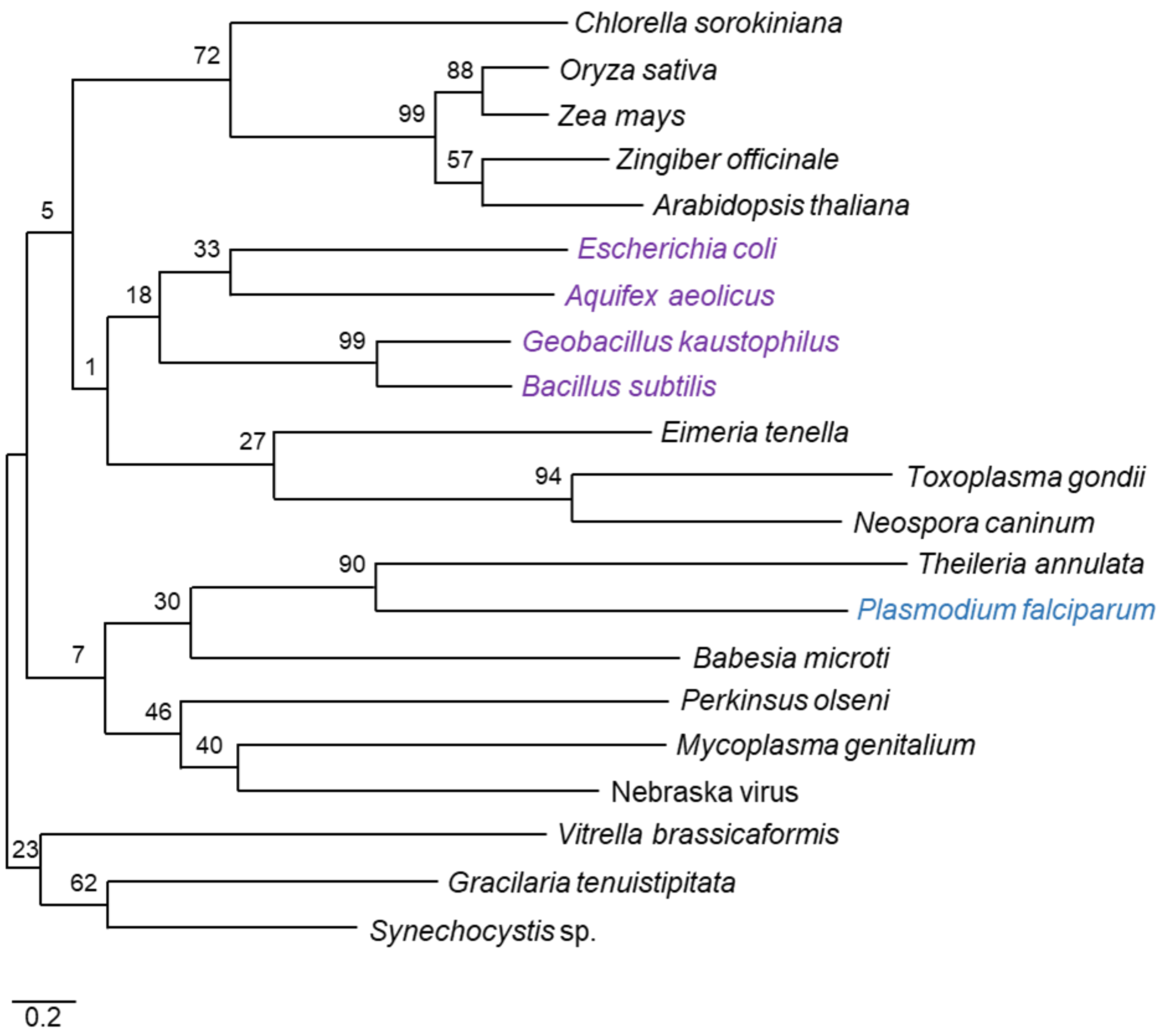

Appendix Figure S3. The phylogenetic relationship among TilS orthologs. Phylogenetic analysis revealed a distant relationship between the TilS proteins of *P. falciparum* and *E. coli*. Bootstrap analyses (1000 replicates) were employed to assess the robustness of the branching patterns. The percentage of replicate trees in which the associated taxa clustered together is indicated adjacent to each branch. The phylogenetic tree is drawn to scale, with branch lengths representing the estimated number of substitutions per site. Refer to **Appendix Table S2** for the accession IDs of the proteins used in this analysis.

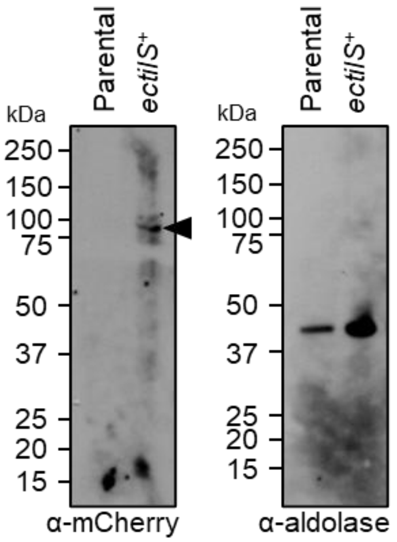

Appendix Figure S4. Uncropped image of immunoblot shown in Fig. 4D. Immunoblot of saponin-isolated $PfMev^{attB}$ (parental) and *ectilS*$^{+}$ parasite lysates with anti-mCherry antibodies (left panel) confirms expression of *Ec*TilS-mCherry fusion protein (expected molecular weight 91 kDa, black arrowhead). Anti-aldolase immunoblot shows relative loading levels (right panel). Protein markers are in kilodaltons (kDa).

```
AfTiaS     -------------------------------------------------------------------------------- 0
PfTilS     MYLLCIFLYILSFLCFTICIKRKQNVSKRWQFFIPHINKEGLYNIMDKKIIRVNKNKRFHRNVVCSNRLSEFIDRDININ 80

AfTiaS     -------------------------------------------------------------------------------- 0
PfTilS     NKMKNIKELKKKHIDNNILIKADIFSNIIEKKNNSNLFFKKFCSFINMEYDETTKDLEELKINKRLKNNNNDNNNNNNNN 160

AfTiaS     -------------------------------------------------------------------------------- 0
PfTilS     MYDNKSNNIHNNNNNNSDNLIKCIKKNQDVLQLVEGYWLLTLKNKYMFSFLKKKKKIIFSVSSGVDSLCLLYSFIFVIYK 240

AfTiaS     --------------------------------------------------------------------------------
PfTilS     ILISMIYKNKSYFGFMNKINSVYSFTHEDIIDIIKEYRYENNVSFFLSILSKIIVIYCHHNTRKECTSEMYFLKNICKKF 320

AfTiaS     -------------------------------------------------------------------------------- 0
PfTilS     GIHFKSKKLTEKSIQKLNVINLKTKKKNYESNVNKKININMMKNKKNFLLLARTWRRNIYVHLSNDILKRDMNNNIYHNN 400

AfTiaS     -------------------------------------------------------------------------------- 0
PfTilS     KMKDSHNNNNNNDNNNTSMKDPLNMLDAYTYEKYIYDINNYIKITNKKCITNVLIKKESFSKEYSNDSIMRKKKKEGLLF 480

                                                            ** *
AfTiaS     -------------------------------------------MRVWVGIDDTDSSRGM--------------------- 16
PfTilS     NNIMNLQNNNIKIINNTCSNNIYNMKYTNVFLNKYCILKKKIKSIVFLGHHQNDNNETVLLQFFRGVFLKNLRGIKFLTY 560

               *                                                  *** *
AfTiaS     ---CTTYLAVLAMER------------------------VERELGKVIGFPRLIRLNPTIPYKTRGNGAVSFLVEVDDVG 69
PfTilS     YKNCLLYRPFIKLNKLHLYRYMQLINKTWNFDSSNNNMSISRNFIRNVVIPNITHMLKDKSYKNRENHNIDKEINEKNDK 640

AfTiaS     ELV------DVVNEVII---------EH------AML-DDEKTNPGAVFVDEELAVKLKPFADKAIKDVLQIDE----AL 123
PfTilS     HMLDDNVCVDYHDETPLHNKKEMNKYDHVEISKNNIVNKNNELKNQHVYVNTSLDRR--------LKNVLRQTTNLENYL 712

                                                                                     *
AfTiaS     FVIGKYFIPHLRHKKGRGLIGALAAVGAELED-----------------------------------------FTLELIA 162
PfTilS     NYYDNMFFTYLKKKYYKRCMSTTKKITHEEETYTNVHDTHNGNIKNKRNITKIHKSYNTYKNSDINLINRNVHLKKNIKC 792

                                                                                     *
AfTiaS     YRYPERFGTEREYDEESFFDMDYELYPQTFDNVDWCN-----------DV---------VVCIPN--TPCPVLYGIRGE- 219
PfTilS     YNYQDA----HNIFMNEYFKMQNNLYNIFFPRYNMETLIKINKKLYEKNIYLKIFNFFELLLLPSKLIRLEILYNIIRKY 868

                                                                                    *
AfTiaS     --------SVEALYKAMESVKTEPVDRRMIFVTNHATDMHLIGEEEVHRLENYRSYRLRGRVTL-EPYDIEGGHVFFEID 290
PfTilS     VKVNIKYAKIERIYEQMIAYINE--------------------------------YIKRDKTKYGTTQSFHQSHIFNDDD 916

                     *
AfTiaS     TKFGSVKCAAFEPTKQFRNVIRLLRKGDVVEVYGSMKKDTINLEKIQIVELAEIWVEKNPICPS---------CGRR--- 358
PfTilS     TMFDNKMRVQNKS-KKEN----KILNVKIKDLFGDTKIEEVNFVVINITKSKSILLQNNLFRIIERDMIEDITKGRKRLH 991

             *       *
AfTiaS     MESAGRGQGFRCKKC-------RTKADEKLR-----EKVE-----R--------------------------ELQ----- 390
PfTilS     MDDNKRDDNKRDDNKRDDNKRDDNKRDDNKRDDNKRDDNKRDDNKRDDNKRDDNKRDDNKRDDNKRDDKKDTHIQHDKDI 1071

AfTiaS     --PGFYEVPPS---------ARRHLSKPLIRMNVEGRHIFR--------------------------------------- 420
PfTilS     TSQTFFPHNKITIANEKCIKNDEYIDNSLVCFKEKSANIFVHNNISTEVSRLKKYDITKKNDKKNIFLLIKKRKKKKKEK 1151

AfTiaS     -------------------------------------------------------------------------------- 420
PfTilS     FHIHIRYIKKNDYVYLEKKKISVNKFLTLHKIPYIYQTALPVIEIINFNNNHILFFYLFPEVKSPYFTLREKTFPQKYMN 1231

AfTiaS     -------------- 420
PfTilS     THFVYSIKFKGIRD 1245
```

* tRNA binding residue   * ATP coordinating residue   CXXC motif

Appendix Figure S5. Sequence alignment between *Archaeoglobus fulgidus* TiaS (*Af*TiaS) and *P. falciparum* TilS (*Pf*TilS). Threonine18 (Bold purple T) of *Af*TiaS is subject to autophosphorylation. Alignment output was visualized using Boxshade to highlight conserved residues. *Af*TiaS and *Pf*TilS accession IDs are provided in the Methods section.

pftilS-3xV5

CCTAGGATGTACCTGCTGTGCATTTTCCTGTATATCTTGAGCTTTTTATGTTTTACAATTTGTATTAAACGTAAACAGAACG
TCTCGAAGCGTTGGCAGTTTTTCATTCCGCATATTAACAAGGAAGGCCTTTACAATATCATGGATAAGAAAATCATCCGGGT
CAACAAAAACAAACGGTTCCATCGTAACGTTGTATGCAGCAACCGGCTGAGTGAATTCATCGATCGCGATATCAACATTAAC
AACAAGATGAAGAATATCAAGGAACTGAAAAAAAAACACATTGATAACAACATTTTGATTAAAGCAGACATTTTCAGCAACA
TAATCGAGAAAAAAAACAATTCAAACTTATTTTTAAAAAATTTTGCTCTTTTATTAACATGGAATATGACGAAACCACCAA
AGATTTAGAAGAGTTAAAAATCAACAAACGATTGAAAAACAATAACAATGATAATAACAACAATAACAACAACAACATGTAT
GATAACAAAAGCAATAATATTCATAACAACAACAATAACAATAGCGATAATTTGATTAAATGTATCAAAAAGAATCAGGATG
TGCTGCAGTTGGTAGAAGGATACTGGCTGTTAACGCTGAAAAACAAATATATGTTTTCTTTTCTGAAAAAAAAGAAAAAAAT
TATCTTCAGCGTGTCAAGTGGGGTGGACTCTTTGTGCCTTCTTTATAGCTTCATCTTTGTTATCTATAAAATTCTGATCAGC
ATGATTTACAAAAATAAAAGTTATTTTGGCTTTATGAATAAAATCAACAGCGTGTATAGCTTTACTCATGAAGATATTATTG
ATATCATCAAAGAATACCGTTATGAAAACAATGTCAGCTTCTTTCTGTCGATCCTGAGCAAAATTATCGTGATTTACTGTCA
TCATAATACCCGTAAAGAATGTACCTCAGAAATGTATTTTCTTAAAAACATTTGCAAAAAATTCGGTATCCACTTTAAATCC
AAAAAACTGACGGAAAAGAGTATTCAGAAACTGAATGTAATAAACTTAAAAACGAAAAAGAAAAATTACGAAAGCAATGTGA
ATAAGAAAATTAATATTAACATGATGAAGAATAAAAAAAATTTCCTGCTGCTAGCACGAACCTGGCGTCGTAACATCTACGT
GCACCTCTCCAACGATATTTTGAAACGGGACATGAACAACAATATTTATCACAACAACAAAATGAAAGATTCCCATAACAAC
AACAACAATAACGATAATAACAACACGAGCATGAAGGACCCCCTGAATATGTTAGATGCTTATACCTACGAAAAATATATTT
ACGATATTAACAATTATATCAAAATCACCAACAAAAAATGCATCACCAACGTCCTGATTAAGAAAGAATCATTCAGTAAGGA
GTACTCCAACGATTCGATCATGCGCAAGAAGAAAAAGGAAGGTCTGCTTTTCAATAATATTATGAACCTGCAGAATAATAAC
ATTAAAATTATCAACAACACTTGCAGCAATAATATTTACAATATGAAATATACTAATGTTTTTCTGAACAAATATTGCATCC
TGAAAAAAAAGATTAAATCAATCGTGTTCCTCGGCCACCACCAGAACGACAACAACGAAACGGTGCTGCTTCAGTTCTTCCG
TGGTGTTTTTTTAAAAAACCTGCGCGGCATTAAATTTCTCACCTACTATAAAAATTGTCTGCTGTATCGTCCGTTCATCAAA
CTGAACAAACTCCATTTGTATCGCTACATGCAGCTTATCAACAAAACTTGGAATTTCGATTCCTCTAACAACAATATGTCTA
TTTCGCGTAACTTCATCCGCAATGTGGTGATCCCAAACATAACCCATATGTTGAAAGATAAAAGCTATAAAAATCGTGAGAA
CCACAATATTGACAAAGAGATTAATGAGAAGAATGATAAGCACATGCTGGATGATAACGTGTGCGTTGATTACCATGATGAA
ACACCCCTTCATAATAAGAAAGAAATGAATAAATATGATCATGTCGAAATTTCGAAAAATAACATCGTTAACAAAAACAATG
AACTGAAAAACCAGCATGTATATGTCAATACCTCTCTGGATCGTCGCCTGAAAAACGTTCTGCGCCAGACCACGAACCTGGA
AAATTACCTGAATTATTATGACAATATGTTCTTTACCTATCTGAAAAAGAAATATTACAAACGCTGCATGTCAACCACAAAA
AAGATTACGCATGAAGAAGAGACGTACACTAACGTCCATGATACCCATAACGGCAACATCAAAAATAAACGCAACATCACCA
AAATTCACAAAAGTTATAACACCTATAAAAACTCCGATATAAATTTAATCAATCGTAACGTGCATTTGAAAAAAAACATTAA
ATGCTACAACTATCAGGATGCGCATAACATCTTTATGAACGAATACTTTAAAATGCAAAACAATCTGTATAACATCTTTTTC
CCGCGTTACAACATGGAAACACTGATCAAAATTAACAAAAAACTGTACGAGAAAAACATTTACCTGAAAATTTTTAACTTCT
TCGAACTCCTGCTGCTGCCTTCTAAACTCATCCGCCTCGAAATTCTGTATAACATCATCCGTAAATATGTCAAGGTAAACAT
TAAATATGCCAAGATAGAACGGATTTATGAACAAATGATTGCGTACATTAATGAATATATCAAACGTGATAAAACAAAATAC
GGTACCACGCAATCGTTTCATCAATCGCATATATTCAACGATGATGATACTATGTTCGACAATAAAATGCGTGTTCAGAATA
AAAGCAAAAAGGAAAATAAAATCCTGAACGTGAAAATTAAGGATTTGTTTGGGGATACCAAAATTGAGGAGGTGAACTTTGT
GGTGATTAATATTACTAAAAGTAAGTCAATCCTTCTCCAAAACAACCTGTTCCGGATTATTGAACGCGATATGATCGAAGAT
ATTACCAAAGGACGCAAACGCCTCCACATGGACGATAACAAACGCGACGATAATAAACGCGACGATAACAAACGCGACGATA
ACAAACGCGACGATAACAAACGGGACGATAATAAACGCGACGATAACAAACGTGACGATAATAAACGTGACGATAATAAACG
CGACGATAACAAACGTGACGACAACAAACGGGACGATAACAAACGGGACGATAATAAACGGGATGATAAAAAAGATACCCAC
ATTCAGCACGATAAGGATATTACATCACAAACCTTTTTCCCTCACAACAAAATTACAATTGCCAACGAGAAATGTATTAAAA
ACGATGAATACATTGACAACAGCCTGGTCTGTTTTAAAGAAAAAAGCGCCAATATCTTTGTCCATAATAATATTTCCACGGA
GGTGTCTCGCCTGAAAAAATATGATATCACGAAAAAGAACGATAAAAAGAACATCTTTCTTCTAATCAAAAAGCGGAAAAAA
AAAAAAAAAGAAAAATTTCACATTCACATTCGATACATTAAAAAAAATGATTATGTTTATCTGGAAAAAAAAAAAATCTCCG
TTAATAAATTTCTGACCCTTCACAAGATCCCGTATATTTATCAGACCGCGCTGCCGGTTATTGAAATTATCAATTTTAATAA
TAACCATATTCTGTTTTTTTATTTGTTCCCGGAAGTGAAAAGCCCGTATTTCACTCTGCGAGAAAAAACGTTTCCACAAAAA
TATATGAATACACATTTTGTTTATAGTATCAAATTCAAGGGGATCCGTGACCGTACGGACGTCGGAAAACCAATACCAAATC
CTTTGTTAGGTTTAGACAGTACAGGAGGTAAACCTATACCTAATCCTTTATTGGGTCTTGATTCAACTGGTGGAAAACCTAT
TCCAAATCCATTATTAGGATTAGATTCTACATAAGGGCCC

Appendix Figure S6. Sequence of codon-modified full-length *pftils* (in blue font) as synthesized. *Avr*II (CCTAGG) and *Psp*OMI (GGGCCC) sites are underlined. The 3xV5 sequence is in magenta.

trpftilS-3xV5

CCTAGGATGATTAAATGTATCAAAAAGAATCAGGATGTGCTGCAGTTGGTAGAAGGATACTGGCTGTTAACGCTGAAAAACA
AATATATGTTTTCTTTTCTGAAAAAAAAGAAAAAAATTATCTTCAGCGTGTCAAGTGGGGTGGACTCTTTGTGCCTTCTTTA
TAGCTTCATCTTTGTTATCTATAAAATTCTGATCAGCATGATTTACAAAAATAAAAGTTATTTTGGCTTTATGAATAAAATC
AACAGCGTGTATAGCTTTACTCATGAAGATATTATTGATATCATCAAAGAATACCGTTATGAAAACAATGTCAGCTTCTTTC
TGTCGATCCTGAGCAAAATTATCGTGATTTACTGTCATCATAATACCCGTAAAGAATGTACCTCAGAAATGTATTTTCTTAA
AAACATTTGCAAAAAATTCGGTATCCACTTTAAATCCAAAAAACTGACGGAAAAGAGTATTCAGAAACTGAATGTAATAAAC
TTAAAAACGAAAAAGAAAAATTACGAAAGCAATGTGAATAAGAAAATTAATATTAACATGATGAAGAATAAAAAAAATTTCC
TGCTGCTAGCACGAACCTGGCGTCGTAACATCTACGTGCACCTCTCCAACGATATTTTGAAACGGGACATGAACAACAATAT
TTATCACAACAACAAAATGAAAGATTCCCATAACAACAACAACAATAACGATAATAACAACACGAGCATGAAGGACCCCCTG
AATATGTTAGATGCTTATACCTACGAAAAATATATTTACGATATTAACAATTATATCAAAATCACCAACAAAAAATGCATCA
CCAACGTCCTGATTAAGAAAGAATCATTCAGTAAGGAGTACTCCAACGATTCGATCATGCGCAAGAAGAAAAAGGAAGGTCT
GCTTTTCAATAATATTATGAACCTGCAGAATAATAACATTAAAATTATCAACAACACTTGCAGCAATAATATTTACAATATG
AAATATACTAATGTTTTTCTGAACAAATATTGCATCCTGAAAAAAAAGATTAAATCAATCGTGTTCCTCGGCCACCACCAGA
ACGACAACAACGAAACGGTGCTGCTTCAGTTCTTCCGTGGTGTTTTTTTAAAAAACCTGCGCGGCATTAAATTTCTCACCTA
CTATAAAAATTGTCTGCTGTATCGTCCGTTCATCAAACTGAACAAACTCCATTTGTATCGCTACATGCAGCTTATCAACAAA
ACTTGGAATTTCGATTCCTCTAACAACAATATGTCTATTTCGCGTAACTTCATCCGCAATGTGGTGATCCCAAACATAACCC
ATATGTTGAAAGATAAAAGCTATAAAAATCGTGAGAACCACAATATTGACAAAGAGATTAATGAGAAGAATGATAAGCACAT
GCTGGATGATAACGTGTGCGTTGATTACCATGATGAAACACCCCTTCATAATAAGAAAGAAATGAATAAATATGATCATGTC
GAAATTTCGAAAAATAACATCGTTAACAAAAACAATGAACTGAAAAACCAGCATGTATATGTCAATACCTCTCTGGATCGTC
GCCTGAAAAACGTTCTGCGCCAGACCACGAACCTGGAAAATTACCTGAATTATTATGACAATATGTTCTTTACCTATCTGAA
AAAGAAATATTACAAACGCTGCATGTCAACCACAAAAAAGATTACGCATGAAGAAGAGACGTACACTAACGTCCATGATACC
CATAACGGCAACATCAAAAATAAACGCAACATCACCAAAATTCACAAAAGTTATAACACCTATAAAAACTCCGATATAAATT
TAATCAATCGTAACGTGCATTTGAAAAAAAACATTAAATGCTACAACTATCAGGATGCGCATAACATCTTTATGAACGAATA
CTTTAAAATGCAAAACAATCTGTATAACATCTTTTTCCCGCGTTACAACATGGAAACACTGATCAAAATTAACAAAAAACTG
TACGAGAAAAACATTTACCTGAAAATTTTTAACTTCTTCGAACTCCTGCTGCTGCCTTCTAAACTCATCCGCCTCGAAATTC
TGTATAACATCATCCGTAAATATGTCAAGGTAAACATTAAATATGCCAAGATAGAACGGATTTATGAACAAATGATTGCGTA
CATTAATGAATATATCAAACGTGATAAAACAAAATACGGTACCACGCAATCGTTTCATCAATCGCATATATTCAACGATGAT
GATACTATGTTCGACAATAAAATGCGTGTTCAGAATAAAAGCAAAAAGGAAAATAAAATCCTGAACGTGAAAATTAAGGATT
TGTTTGGGGATACCAAAATTGAGGAGGTGAACTTTGTGGTGATTAATATTACTAAAAGTAAGTCAATCCTTCTCCAAAACAA
CCTGTTCCGGATTATTGAACGCGATATGATCGAAGATATTACCAAAGGACGCAAACGCCTCCACATGGACGATAACAAACGC
GACGATAATAAACGCGACGATAACAAACGCGACGATAACAAACGCGACGATAACAAACGGGACGATAATAAACGCGACGATA
ACAAACGTGACGATAATAAACGTGACGATAATAAACGCGACGATAACAAACGTGACGACAACAAACGGGACGATAACAAACG
GGACGATAATAAACGGGATGATAAAAAAGATACCCACATTCAGCACGATAAGGATATTACATCACAAACCTTTTTCCCTCAC
AACAAAATTACAATTGCCAACGAGAAATGTATTAAAAACGATGAATACATTGACAACAGCCTGGTCTGTTTTAAAGAAAAAA
GCGCCAATATCTTTGTCCATAATAATATTTCCACGGAGGTGTCTCGCCTGAAAAAATATGATATCACGAAAAAGAACGATAA
AAAGAACATCTTTCTTCTAATCAAAAAGCGGAAAAAAAAAAAAAAAAGAAAAATTTCACATTCACATTCGATACATTAAAAAA
AATGATTATGTTTATCTGGAAAAAAAAAAAAATCTCCGTTAATAAATTTCTGACCCTTCACAAGATCCCGTATATTTATCAGA
CCGCGCTGCCGGTTATTGAAATTATCAATTTTAATAATAACCATATTCTGTTTTTTTATTTGTTCCCGGAAGTGAAAAGCCC
GTATTTCACTCTGCGAGAAAAAACGTTTCCACAAAAATATATGAATACACATTTTGTTTATAGTATCAAATTCAAGGGGATC
CGTGACCGTACGGACGTCGGAAAACCAATACCAAATCCTTTGTTAGGTTTAGACAGTACAGGAGGTAAACCTATACCTAATC
CTTTATTGGGTCTTGATTCAACTGGTGGAAAACCTATTCCAAATCCATTATTAGGATTAGATTCTACATAAGGGCCC

Appendix Figure S7. Sequence of codon-modified N-terminally truncated *pftils* (in blue font) as synthesized. *Avr*II (CCTAGG) and *Psp*OMI (GGGCCC) sites are underlined. The 3xV5 sequence is in magenta.

Appendix Table S1. Primers used in this study. Restriction enzyme sites are underlined.

Primer name	Sequence (5'→3')	Primer description
Primers to amplify homology arms (HA) and guide RNA (gRNA) annealing for *pftilS* knockout		
TilS.HA1F	GTGCCACGAGCGGCCGCAGGTTCCATAGAAATGTTGTGTG	Forward for HA1 amplification
TilS.HA1R	AAGCGCAGCGGCCGCGTAAAACATCTTGATTCTTTTTAATACA	Reverse for HA1 amplification
TilS.HA2F	TTCGACAGACGCCGGCAAAGGATATATGAACAGATGATAGCA	Forward for HA2 amplification
TilS.HA2R	TGGCCACCAGCCGGCATCATGTTGTATATGTGTATCTTTTT	Reverse for HA2 amplification
Tils.gRNA.pF	/5Phos/TATTTCAAGATGTTTTACAACTTG	5'-phosphorylated guide RNA forward oligo
Tils.gRNA.pR	/5Phos/AAACCAAGTTGTAAAACATCTTGA	5'-phosphorylated guide RNA reverse oligo
Primers for *pftilS* gene knockout confirmation		
TilS.5.F	ATGTGTCGAAGAGATGGCAATTTTTCATACCACAT	Forward for 5' and Δ5' PCR
TilS.5.WT.R	TATGAATGAATAAAGTAGACACAAAGAATCTACCCCTGA	Reverse for 5' PCR
TilS.3.WT.F	TGAACTTTTATTATTACCCTCTAAACTAATTAGACTAGAAAT	Forward for 3' PCR
TilS.3.R	TACAAAAATGTTAGCACTTTTTTCTTTAAAGCACACTAATG	Reverse for 3' and Δ3' PCR
pRS.F	CATATTTATTAAATCTAGAATTCGACAGACGCCG	Forward for Δ3' PCR
pRS.R	TACAAAATGCTTAAGCGCAGCGGCC	Reverse for Δ5' PCR

Primers to amplify representative genes from nuclear and organellar genome		
LDH.F	GGAGATGTAGTTTTGTTCGATATTG	Forward for PCR
LDH.R	CTTGTAAAGGGATACCACCTACAG	Reverse for PCR
SufB.F	CATGTAGCTATAGTAGAAATAATAGTAAAAGATTATGG	Forward for PCR
SufB.R	GACTCTGAAATACTTAAACCACGTTGC	Reverse for PCR
Cox1.F	CTTCATCTTTAAGAATAATTGCACAAGAAAATGTAAATC	Forward for PCR
Cox1.R	GTACATATGATGTACCCATACTAAGCTTCC	Reverse for PCR
Primers for generation of pCLD-*ectilS-mcherry-10xapt* plasmid		
EcTils.InF F	GTTAGAAGGTTCCGGAATGACACTCACGCTCAATAGACAAC	Forward for *ectilS* amplification
EcTils.InF R	GCCCTTGCTCGTACGACTAAGCGTTTTCTGCCAGACAAAAC	Reverse for *ectilS* amplification
Primers for confirmation of gene knock-in		
P230p.out.HA.F	GGTTGTGATTTTTCAGGTGATTCC	Forward for attL and attB PCR
P230p.out.HA.R	GAAAATTGTAGGGGCAGCTAAATCCGAC	Reverse for attR and attB PCR
attB.Int.F	GCAGTGTGGAATTCCCTGCA	Reverse for attL PCR
attB.Int.R	TTAAGTGTAGTTAATTCATCAAATAGCATGC	Forward for attR PCR
Primers for sequencing		
pRS.R	TACAAAATGCTTAAGCGCAGCGGCC	For HA1 insertion in pRSng-*pftilS*

pRS.F	CATATTTATTAAATCTAGAATTCGACAGACGCCG	For HA2 insertion in pRSng-*pftilS*
pL6.gRNA.F	GGGTAAATTATTATTAAAAAATGTATATGTTATG	For guide RNA insertion in pCasG-LacZ
CLD2.F	ACAACCTAGGATGAAGATCTTATTAC	Forward for *ectilS* insertion in pCLD-*ectilS-mcherry-10xapt*
RFP.R	GAGGGCTCCGTGAACGGC	Reverse for *ectilS* insertion in pCLD-*ectilS-mcherry-10xapt*
Cre.Ins.Seq.F	CAACCTAGATAACTTCGTATAGCATACATTATACG	Forward for *pftilS* (full-length or truncated) insertion in pCre-*tr/pftilS-3xV5*
TilS.Seq.F1	GGATGTGCTGCAGTTGGTAGAAGGA	Forward for *pftilS* (full-length or truncated) insertion in pCre-*tr/pftilS-3xV5*
TilS.Seq.F2	CAGTAAGGAGTACTCCAACGATTCG	Forward for *pftilS* (full-length or truncated) insertion in pCre-*tr/pftilS-3xV5*
TilS.Seq.F3	GACCACGAACCTGGAAAATTACC	Forward for *pftilS* (full-length or truncated) insertion in pCre-*tr/pftilS-3xV5*
NewApt.5R	CTCGCTATCAAGGAATCGAGTCC	Reverse for *pftilS* (full-length or truncated) insertion in pCre-*tr/pftilS-3xV5*

Appendix Table S2. Proteins used for phylogenetic analysis presented in Appendix Fig. S3. [a]Uniprot ID, [b]PlasmoDB ID, [c]ToxoDB ID, [d]PiroplasmaDB ID.

Species	Accession	Gene name
Only Syngen Nebraska virus 5 (Nv) (Chlorovirus)	A0A1J0F9X6[a]	OS5_287L
Chlorella sorokiniana (Freshwater green alga)	A0A2P6TQ55[a]	C2E21_4934
Gracilaria tenuistipitata var. *liui* (Red alga)	Q6B8L1[a]	tilS, ycf62, Grc000193
Oryza sativa (Monocot)	B8AFB7[a]	OsI_06667
Zea mays (Monocot)	A0A804PGD6[a]	tilS
Zingiber officinale (Monocot root plant)	A0A8J5GGP9[a]	ZIOFF_041066
Arabidopsis thaliana (Dicot)	F4J7P7[a]	RSY3
Perkinsus olseni (Dinoflagellate)	A0A7J6UN02[a]	FOZ63_029171
Synechocystis sp, PCC 6803 (Freshwater cyanobacteria)	P74192[a]	tilS, slr1278
Vitrella brassicaformis CCMP3155 (Dinoflagellate)	A0A0G4FYP9[a]	Vbra_16478
Escherichia coli (Gram-negative bacteria)	P52097[a]	tilS, mesJ
Geobacillus kaustophilus (Thermophilic Gram-positive bacteria)	Q5L3T3[a]	tilS, GK0060
Bacillus subtilis (Gram-positive bacteria)	P37563[a]	tilS, yacA
Aquifex aeolicus (chemolithoautotrophic Gram-negative bacteria)	O67728[a]	tilS, aq_1887
Mycoplasma genitalium (Gram-negative bacteria)	P47330[a]	tilS, MG084
Plasmodium falciparum (Apicomplexan)	PF3D7_0411200[b]	tilS, PP-loop family protein
Toxoplasma gondii (Apicomplexan)	TGME49_215100[c]	tilS, PP-loop family protein
Neospora caninum (Apicomplexan)	NCLIV_052110[c]	tilS, hypothetical protein

Eimeria tenella (Apicomplexan)	ETH2_0718500[c]	tilS, PP-loop family protein
Babesia microti (Apicomplexan)	BMR1_01G01110[d]	tilS, PP-loop family protein
*Theileria annulata (*Apicomplexan)	TA03600[d]	tilS, hypothetical protein

Appendix Table S3. tRNAs used for phylogenetic analysis presented in Fig. EV2. Accession ID for apicoplast-genome-encoded tRNAs are provided. *Pfal*, *Plasmodium falciparum*; *Tgon*, *Toxoplasma gondii*; *Eten*, *Eimeria tenella*; *Bmic*, *Babesia microti*; *Tpar*, *Theileria parva*; *Hmar*, *Haloarcula marismortui*; *Mmob*, *Mycoplasma mobile*; *Bsub*, *Bacillus subtilis*; *Ecol*, *Escherichia coli*; *Cpan*, *Cycas panzhihuaensis*; *Slyc*, *Solanum lycopersicum*; *Scer*, *Saccharomyces cerevisiae*.

tRNA	Accession ID	Sequence (5' → 3')
*Pfal*_trnM-CAU1	PF3D7_API06600	AGCGAAAUAGAGCAUAAGGAAAGUUCGUCGGAUUCAUGCUCCGAAGGUAAUCGGUUCAAUUCCGUUUUUCGCUUA
*Pfal*_trnM-CAU2	PF3D7_API00600	AACAUUUAUAGCUAAGUGGUCGAAAGCAAUGGACUCAUAAUUCAUUUUCAUAUAUUGAUCAUCAGUAGUUCGAAUCUACUUAAAUGU
*Pfal*_trnM-CAU3	PF3D7_API05000	AGCGAAAUAGAGCAUAAGGAAAGUUCGUCGGAUUCAUGCUCCGAAGGUAAUCGGUUCAAUUCCGUUUUUCGCUU
*Pfal*_trnI-GAU	PF3D7_API05800	AUAGGUUUUUAGUUUAAUGGUUAAAACAUACUCUUGAUAAGGGUAAAAUUUUAGUUCAAUUCUAAAAUAACC
*Tgon*_trnM-CAU1	TGME49_355180	AGCGGGGUAGAGCAGGUUGGUAGCUCGUCGGGCUCAUGACCCGAAGGUCAGCGGUUCAAAUCCGCUCCUCGUUU
*Tgon*_trnM-CAU2	TGME49_355060	AUACUUGUGGCUGAGUGGGCAAAAGCAGUGAGCUCAUAACUCAUAUAAAACGAAAGUUCGAAUCUUUUCAAGUAUA
*Tgon*_trnI-GAU1	TGME49_355100	AGGCUAGUAGCUCAACGGUAGAGCACGCUUUUGAUAAGGGCGUGGUUUCUGGUUCGAUUCCAGGGUGGCCUA
*Tgon*_trnI-GAU2	TGME49_355110	AGGCUAGUAGCUCAACGGUAGAGCACGCUUUUGAUAAGGGCGUGGUUUCUGGUUCGAUUCCAGGGUGGCCUA
*Eten*_trnM-CAU1	ETH2_API00800	AAACGGAGUAGAGCAGUCUGGUUAGCUCAUCGGGCUCAUGAUCCGAAGGUCAACGGUUCAAUUCCGUUCUCCGUUUU
*Eten*_trnM-CAU2	ETH2_API01500	UGUACCUGUGGCUGAGUGGUCAAAAGCGGUGGGCUCAUAAUCCAUUUUUUUUCAAAAGUUCAAAUCUUUUCAGGUAUAA
*Eten*_trnM-CAU3	ETH2_API05600	AAACGGAGUAGAGCAGUCUGGUUAGCUCAUCGGGCUCAUGAUCCGAAGGUCAACGGUUCAAUUCCGUUCUCCGUUUU

*Eten*_trnI-GAU1	ETH2_API00100	GGGCUGUUAGCUCAUCGGUAGAGCGCGCCCCUGAUAAGGGCGAGGUACCUGGUUCAACCCCAGGACGGCCUA
*Eten*_trnI-GAU2	ETH2_API06300	GGGCUGUUAGCUCAUCGGUAGAGCGCGCCCCUGAUAAGGGCGAGGUACCUGGUUCAACCCCAGGACGGCCUA
*Bmic*_trnM-CAU1	BmR1_api00060	AAUAAGAUAUAGUAAUAAGGAAACUUACCAGCUUCAUGGUCUGGAGAUUGCAGUUCGAGUCUGCAUCUUAUUU
*Bmic*_trnM-CAU2	BmR1_api00070	AUAUCUGUAGCUUAGUGGUUAUAGCAAUGGGCCCAUGACUCAUUAAUUUCAGUAGUUCAAAUCUACUCAGAUAUA
*Bmic*_trnM-UAU	BmR1_api00200	AAUAAUUUAUUAUUUUAUAUAUAUAUAUUAUAUAUAUAUAAAAUAAUAAAUAUUU
*Bmic*_trnI-GAU	BmR1_api00090	AGAUUUUUAGUUUACUGGUAAAACAUAUCUUUGAUAAGGAUAAAAUAUUUGGUUCAAUUCCAAAAAAAUCUA
*Tpar*_trnM-CAU	TpMuguga_05g00051	GUAUCUAUAGCUUAGAGGCUAAAGCGAUGAGUUCAUACCUCAUGUACAGUAGUUCAAAUCUACUUAGAUAUA
*Tpar*_trnI-GAU	TpMuguga_05g00069	GGACUUUUAGCUUAAUUGUUAAAGUUUACAUGUGAUAUAUGUGAGAGUUUUGGUUAAAAUCCAAAAAAGUCCA
*Hmar*_trnM-CAU		GCCCGGGUGGCUUAGCUGGACAUAGCGCCGCACUCAUAAUGCGGAGAUCGAGGGUUCGGA
*Hmar*_trnfM-CAU		AGCGGGAUGGGAUAGCCAGGAGAUUCCGGCGGGCUCAUAACCCGCAGAUCGGUAGUUCAAAUCUACCUCCCGCUA
*Hmar*_trnI-CAU		GGGCCCUUAGCUUAGUCUGGUUAAAGCGAUCGGCUCAUAACCGAUUGAGCGCUGGUUCAAAUCCGGCAGGGCCCA
*Hmar*_trnI-GAU		GGGCCAAUAGCUCAAUCAGGUUGAGCGCUCGGCUGAUAACCGGGAGGUUCGCGGUUCAAAUCCGCGUUGGCCCA
*Mmob*_trnM-CAU		GGCUCUGUAGCUCAGCUGGUUAGAGCAUUCGGUUCAUACCCGAAAGGUCAAGAGUUCGACUCUCUUCGGAGCUACCA
*Mmob*_trnI-GAU		GGGAGCGUAGCUCAGCUGGUUAGAGCACACGACUGAUAAUCGUGAGGUCGAUGGUUCGAGUCCAUUCGUUCCCACCA

*Mmob*_trnI-UAU		GGUCCUAUAGCUCAGUCGGUUAGAGCACACGACUUAUAAUCGUGAGGUCGCUGGUUCAAUCCCAGCUAGGACUACCA
*Bsub*_trnM-CAU1		GGCGGUGUAGCUCAGCUGGCUAGAGCGUACGGUUCAUACCCGUGAGGUCGGGGGUUCGAUCCCCUCCGCCGCUACCA
*Bsub*_trnM-CAU2		GGCGGUGUAGCUCAGCUGGCUAGAGCGUACGGUUCAUACCCGUGAGGUCGGGGGUUCGAUCCCCUCCGCCGCUACCA
*Bsub*_trnfM-CAU1		CGCGGGGUGGAGCAGUUCGGUAGCUCGUCGGGCUCAUAACCCGAAGGUCGCAGGUUCAAAUCCUGCCCCCGCAACCA
*Bsub*_trnfM-CAU2		CGCGGGGUGGAGCAGUUCGGUAGCUCGUCGGGCUCAUAACCCGAAGGUCGCAGGUUCAAAUCCUGCCCCCGCAACCA
*Bsub*_trnfM-CAU3		CGCGGGGUGGAGCAGUUCGGUAGCUCGUCGGGCUCAUAACCCGAAGGUCGCAGGUUCAAAUCCUGCCCCCGCAACCA
*Bsub*_trnI2-CAU1		GGACCUUUAGCUCAGUUGGUUAGAGCAGACGGCUCAUAACCGUCCGGUCGUAGGUUCGAGUCCUACAAGGUCCACCA
*Bsub*_trnI-GAU1		GGGCCUGUAGCUCAGCUGGUUAGAGCGCACGCCUGAUAAGCGUGAGGUCGAUGGUUCGAGUCCAUUCAGGCCCACCA
*Bsub*_trnI2-GAU1		GGGCCUGUAGCUCAGCUGGUUAGAGCGCACGCCUGAUAAGCGUGAGGUCGGUGGUUCGAGUCCACUCAGGCCCACCA
*Bsub*_trnI2-GAU2		GGGCCUGUAGCUCAGCUGGUUAGAGCGCACGCCUGAUAAGCGUGAGGUCGGUGGUUCGAGUCCACUCAGGCCCACCA
*Ecol*_trnfM-CAU1		CGCGGGGUGGAGCAGCCUGGUAGCUCGUCGGGCUCAUAACCCGAAGGUCGUCGGUUCAAAUCCGGCCCCCGCAACCA
*Ecol*_trnfM-CAU2		CGCGGGGUGGAGCAGCCUGGUAGCUCGUCGGGCUCAUAACCCGAAGGUCGUCGGUUCAAAUCCGGCCCCCGCAACCA
*Ecol*_trnfM-CAU3		CGCGGGGUGGAGCAGCCUGGUAGCUCGUCGGGCUCAUAACCCGAAGGUCGUCGGUUCAAAUCCGGCCCCCGCAACCAA

*Ecol*_trnfM-CAU4		CGCGGGGUGGAGCAGCCUGGUAGCUCGUCGGGCUCAUAACCCGAAGAUCGUCGGUUCAAAUCCGGCCCCCGCAACCA
*Ecol*_trnM-CAU1		GGCUACGUAGCUCAGUUGGUUAGAGCACAUCACUCAUAAUGAUGGGGUCACAGGUUCGAAUCCCGUCGUAGCCACCA
*Ecol*_trnM-CAU2		GGCUACGUAGCUCAGUUGGUUAGAGCACAUCACUCAUAAUGAUGGGGUCACAGGUUCGAAUCCCGUCGUAGCCACCA
*Ecol*_trnI-GAU1		AGGCUUGUAGCUCAGGUGGUUAGAGCGCACCCCUGAUAAGGGUGAGGUCGGUGGUUCAAGUCCACUCAGGCCUACCA
*Ecol*_trnI-GAU2		AGGCUUGUAGCUCAGGUGGUUAGAGCGCACCCCUGAUAAGGGUGAGGUCGGUGGUUCAAGUCCACUCAGGCCUACCA
*Ecol*_trnI2-CAU1		GGCCCCUUAGCUCAGUGGUUAGAGCAGGCGACUCAUAAUCGCUUGGUCGCUGGUUCAAGUCCAGCAGGGGCCACCA
*Ecol*_trnI2-CAU2		GGCCCUUUAGCUCAGUGGUUAGAGCAGGCGACUCAUAAUCGCUUGGUCGCUGGUUCAAGUCCAGCAAGGGCCACCA
*Ecol*_trnI-GAU3		AGGCUUGUAGCUCAGGUGGUUAGAGCGCACCCCUGAUAAGGGUGAGGUCGGUGGUUCAAGUCCACUCAGGCCUACCA
*Cpan*_trnM-CAU1		ACCUACUUAACUCAGUGGUUAGAGUAUCGCUUUCAUACGGCGGGAGUCAUUGGUUCAAAUCCAAUAGUAGGUA
*Cpan*_trnM-CAU2		UGCGGGGUAGAGCAGUUUGGUAGCUCGCAAGGCUCAUAACCUUGAGGUCACGGGUUCAAAUCCCGUCUCCGCCA
*Cpan*_trnI-GAU1		UGGGCUAUCCUGGACUUGAACCAGAGACCUCGCCCGUAUCAGGGGCGCGCUCUACCACUGAGCUAAUAGCCC
*Cpan*_trnI-GAU2		GGGCUAUUAGCUCAGUGGUAGAGCGCGCCCCUGAUGGGCGAGGUCUCUGGUUCAAGUCCAGGAUAGCCCA
*Cpan*_trnI-AUA		GCAUCCAUGGCUGAACGGUUAAAGCGCCCAACUCAUAAUUGGCGAAUUCGCAGGUUCAAUUCCUGCUGGAUGCA

*Slyc*_trnfM-CAU		CGCGGGGUAGAGCAGUUUGGUAGCUCGCAAGGCUCAUAACCUUGAGGUCACGGGUUCAAAUCCUGUCUCCGCAA
*Slyc*_trnM-CAU		ACCUACUUAACUCAGUGGUUAGAGUACUGCUUUCAUACGGCGGGAGUCAUUGGUUCAAAUCCAAUAGUAGGUA
*Slyc*_trnI-CAU		GCAUCCAUGGCUGAAUGGUUAAAGCGCCCAACUCAUAAUUGGCGAAUUCGUAGGUUCAAUUCCUACUGGAUGCA
*Slyc*_trnI-GAU		GGGCUAUUAGCUCAGUGGUAGAGCGCGCCCCUGAUAAUUGCGGGGCGAGGUCUCUGGUUCAAGUCCAGGAUGGCCCA
*Scer*_trniM-CAU1		AGCGCCGUGGCGCAGUGGAAGCGCGCAGGGCUCAUAACCCUGAUGUCCUCGGAUCGAAACCGAGCGGCGCUA
*Scer*_trniM-CAU2		AGCGCCGUGGCGCAGUGGAAGCGCGCAGGGCUCAUAACCCUGAUGUCCUCGGAUCGAAACCGAGCGGCGCUA
*Scer*_trniM-CAU3		AGCGCCGUGGCGCAGUGGAAGCGCGCAGGGCUCAUAACCCUGAUGUCCUCGGAUCGAAACCGAGCGGCGCUA
*Scer*_trniM-CAU4		AGCGCCGUGGCGCAGUGGAAGCGCGCAGGGCUCAUAACCCUGAUGUCCUCGGAUCGAAACCGAGCGGCGCUA
*Scer*_trniM-CAU5		AGCGCCGUGGCGCAGUGGAAGCGCGCAGGGCUCAUAACCCUGAUGUCCUCGGAUCGAAACCGAGCGGCGCUA
*Scer*_trnM-CAU1		GCUUCAGUAGCUCAGUAGGAAGAGCGUCAGUCUCAUAAUCUGAAGGUCGAGAGUUCGAACCUCCCCUGGAGCA
*Scer*_trnM-CAU2		GCUUCAGUAGCUCAGUAGGAAGAGCGUCAGUCUCAUAAUCUGAAGGUCGAGAGUUCGAACCUCCCCUGGAGCA
*Scer*_trnM-CAU3		GCUUCAGUAGCUCAGUAGGAAGAGCGUCAGUCUCAUAAUCUGAAGGUCGAGAGUUCGAACCUCCCCUGGAGCA
*Scer*_trnM-CAU4		GCUUCAGUAGCUCAGUAGGAAGAGCGUCAGUCUCAUAAUCUGAAGGUCGAGAGUUCGAACCUCCCCUGGAGCA

*Scer*_trnM-CAU5		GCUUCAGUAGCUCAGUAGGAAGAGCGUCAGUCUCAUAAUCUGAAGGUCGAGAGUUCGAACCUCCCCUGGAGCA
*Scer*_trnM-CAU6		UGCAAUAUGAUGUAAUUGGUUAACAUUUUAGGGUCAUGACCUAAUUAUAUACGUUCAAAUCGUAUUAUUGCUA
*Scer*_trnM-CAU7		GCUUGUAUAGUUUAAUUGGUUAAAACAUUUGUCUCAUAAAUAAAUAAUGUAAGGUUCAAUUCCUUCUACAAGUA
*Scer*_trnI-AAU1		GGUCUCUUGGCCCAGUUGGUUAAGGCACCGUGCUAAUAACGCGGGGAUCAGCGGUUCGAUCCCGCUAGAGACCA
*Scer*_trnI-AAU2		GGUCUCUUGGCCCAGUUGGUUAAGGCACCGUGCUAAUAACGCGGGGAUCAGCGGUUCGAUCCCGCUAGAGACCA
*Scer*_trnI-AAU4		GGUCUCUUGGCCCAGUUGGUUAAGGCACCGUGCUAAUAACGCGGGGAUCAGCGGUUCGAUCCCGCUAGAGACCA
*Scer*_trnI-AAU3		GGUCUCUUGGCCCAGUUGGUUAAGGCACCGUGCUAAUAACGCGGGGAUCAGCGGUUCGAUCCCGCUAGAGACCA
*Scer*_trnI-AAU5		GGUCUCUUGGCCCAGUUGGUUAAGGCACCGUGCUAAUAACGCGGGGAUCAGCGGUUCGAUCCCGCUAGAGACCA
*Scer*_trnI-AAU6		GGUCUCUUGGCCCAGUUGGUUAAGGCACCGUGCUAAUAACGCGGGGAUCAGCGGUUCGAUCCCGCUAGAGACCA
*Scer*_trnI-AAU7		GGUCUCUUGGCCCAGUUGGUUAAGGCACCGUGCUAAUAACGCGGGGAUCAGCGGUUCGAUCCCGCUAGAGACCA
*Scer*_trnI-AAU9		GGUCUCUUGGCCCAGUUGGUUAAGGCACCGUGCUAAUAACGCGGGGAUCAGCGGUUCGAUCCCGCUAGAGACCA
*Scer*_trnI-AAU8		GGUCUCUUGGCCCAGUUGGUUAAGGCACCGUGCUAAUAACGCGGGGAUCAGCGGUUCGAUCCCGCUAGAGACCA
*Scer*_trnI-AAU10		GGUCUCUUGGCCCAGUUGGUUAAGGCACCGUGCUAAUAACGCGGGGAUCAGCGGUUCGAUCCCGCUAGAGACCA

*Scer*_trnI-AAU11		GGUCUCUUGGCCCAGUUGGUUAAGGCACCGUGCUAAUAACGCGGGGAUCAGCGGUUCGAUCCCGCUAGAGACCA
*Scer*_trnI-AAU12		GGUCUCUUGGCCCAGUUGGUUAAGGCACCGUGCUAAUAACGCGGGGAUCAGCGGUUCGAUCCCGCUAGAGACCA
*Scer*_trnI-AAU13		GGUCUCUUGGCCCAGUUGGUUAAGGCACCGUGCUAAUAACGCGGGGAUCAGCGGUUCGAUCCCGCUAGAGACCA
*Scer*_trnI-GAU		GAAACUAUAAUUCAAUUGGUUAGAAUAGUAUUUUGAUAAGGUACAAAUAUAGGUUCAAUCCCUGUUAGUUUCA
*Scer*_trnI-UAU1		GCUCGUGUAGCUCAGUGGUUAGAGCUUCGUGCUUAUAGCAACAUUCGGUUUCCGAAGUUUCUGUGCCAAAGACCUUUCAAACAGGCCUUUAAAAGCAACGCGACCGUCGUGGGUUCAAACCCCACCUCGAGCA
*Scer*_trnI-UAU2		GCUCGUGUAGCUCAGUGGUUAGAGCUUCGUGCUUAUAGCAACAUUCGGUUUCCGAAGUUUCUGUGCCAAAGACCUUUCAAACAGGCCUUUAAAAGCAACGCGACCGUCGUGGGUUCAAUCCCCACCUCGAGCA
