# Supplementary figures and images for: tRNA lysidinylation is essential for the minimal translation system in the Plasmodium falciparum apicoplast
Source: EMBO Rep. 2025 Mar 20;26(9):2300–22. doi: 10.1038/s44319-025-00420-w (PMC12069591; doi:10.1038/s44319-025-00420-w)

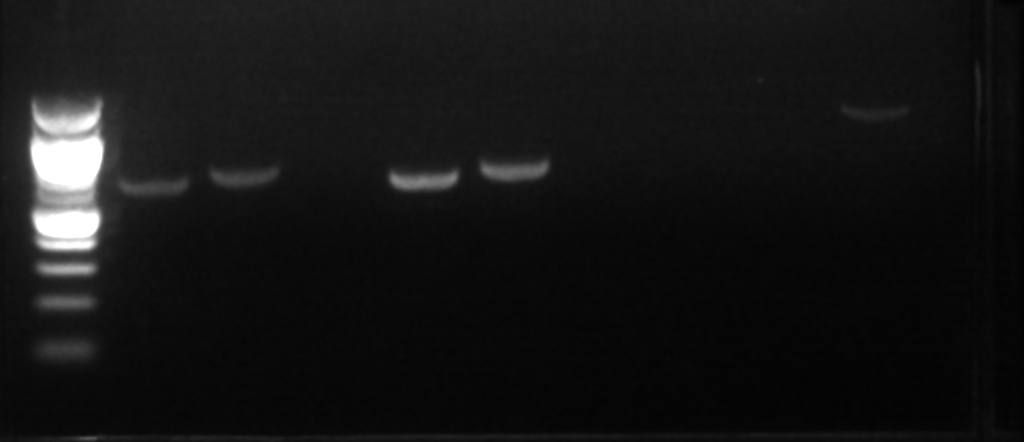

Supplement: Supplementary file 3 — Source data Fig. 2 [file 44319_2025_420_MOESM3_ESM.zip › Figure 2/2C/Fig 2C_genotyping PCR.tif]

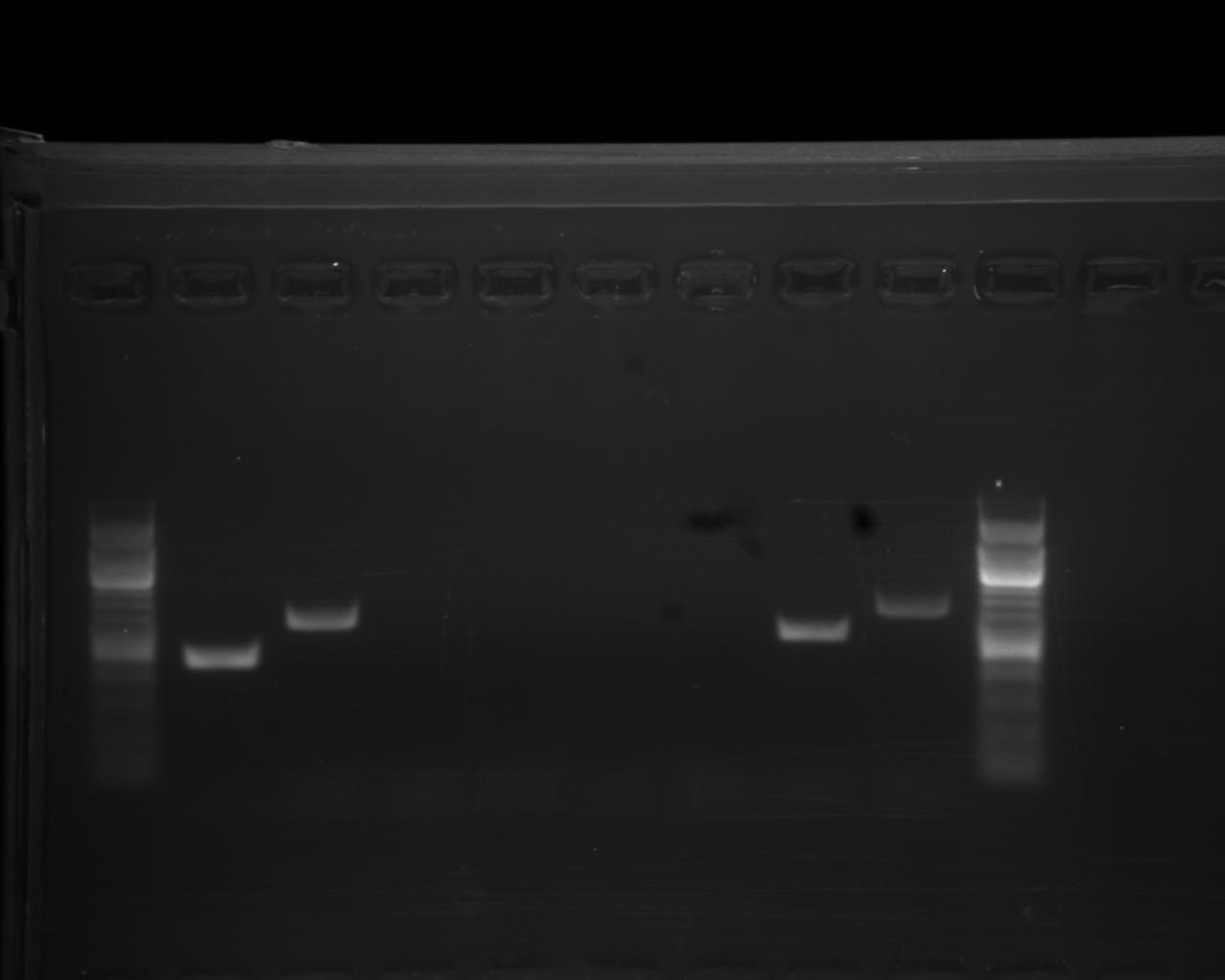

Supplement: Supplementary file 4 — Source data Fig. 3 [file 44319_2025_420_MOESM4_ESM.zip › Figure 3/3B/Fig 3B_genotyping PCR.tif]

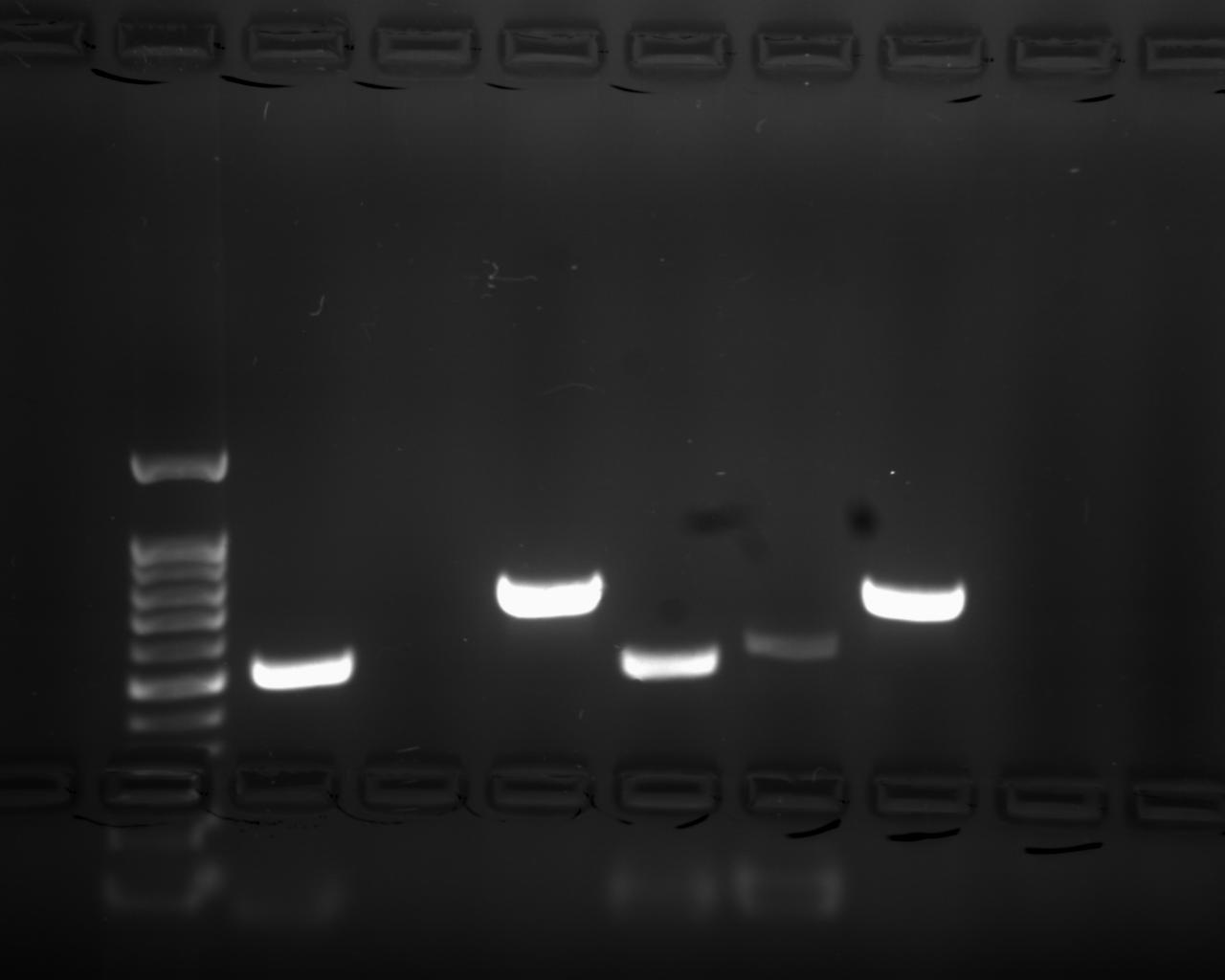

Supplement: Supplementary file 4 — Source data Fig. 3 [file 44319_2025_420_MOESM4_ESM.zip › Figure 3/3C/Fig 3C_NAM PCR.tif]

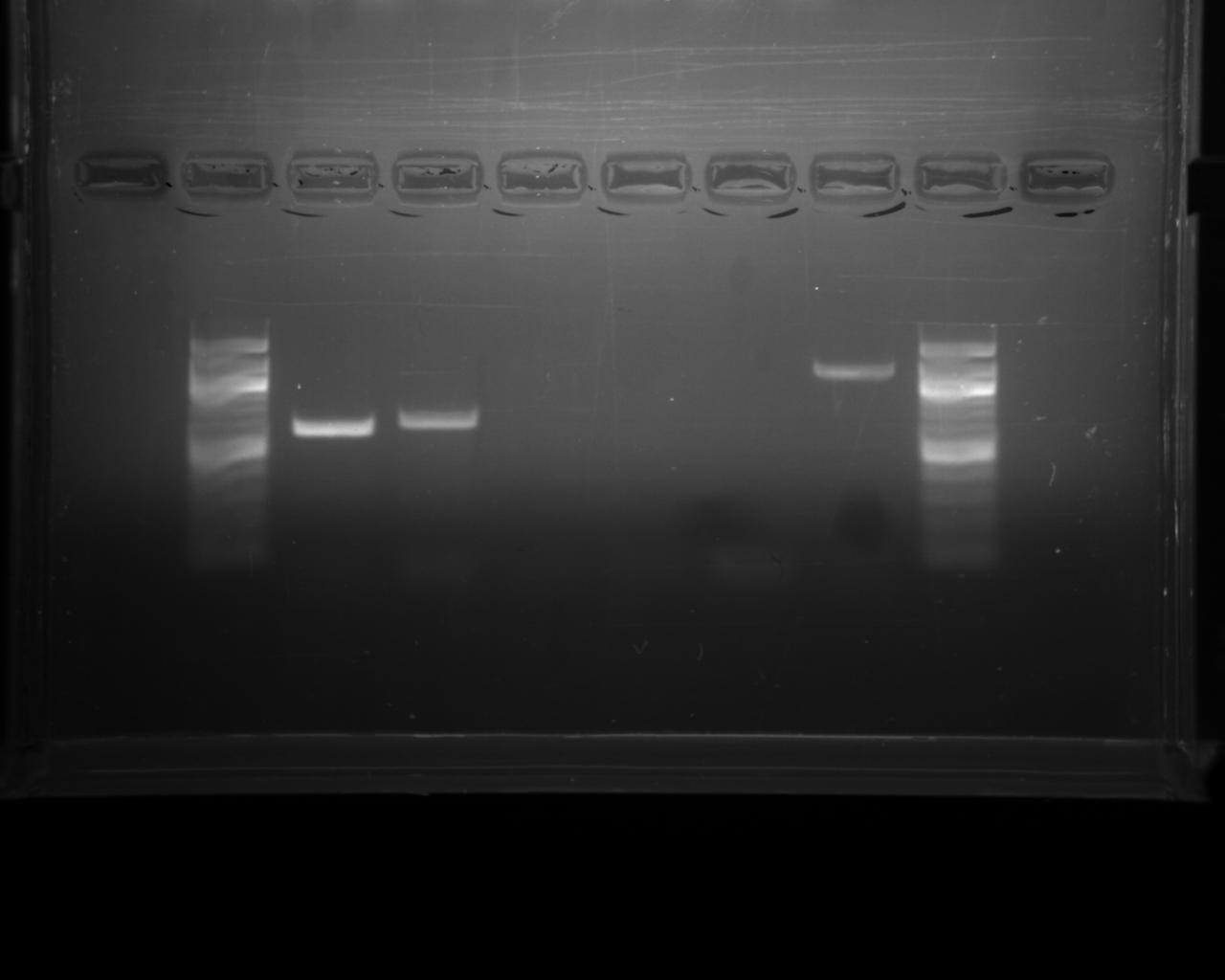

Supplement: Supplementary file 5 — Source data Fig. 4 [file 44319_2025_420_MOESM5_ESM.zip › Figure 4/4C/Fig 4C_genotyping PCR.tif]

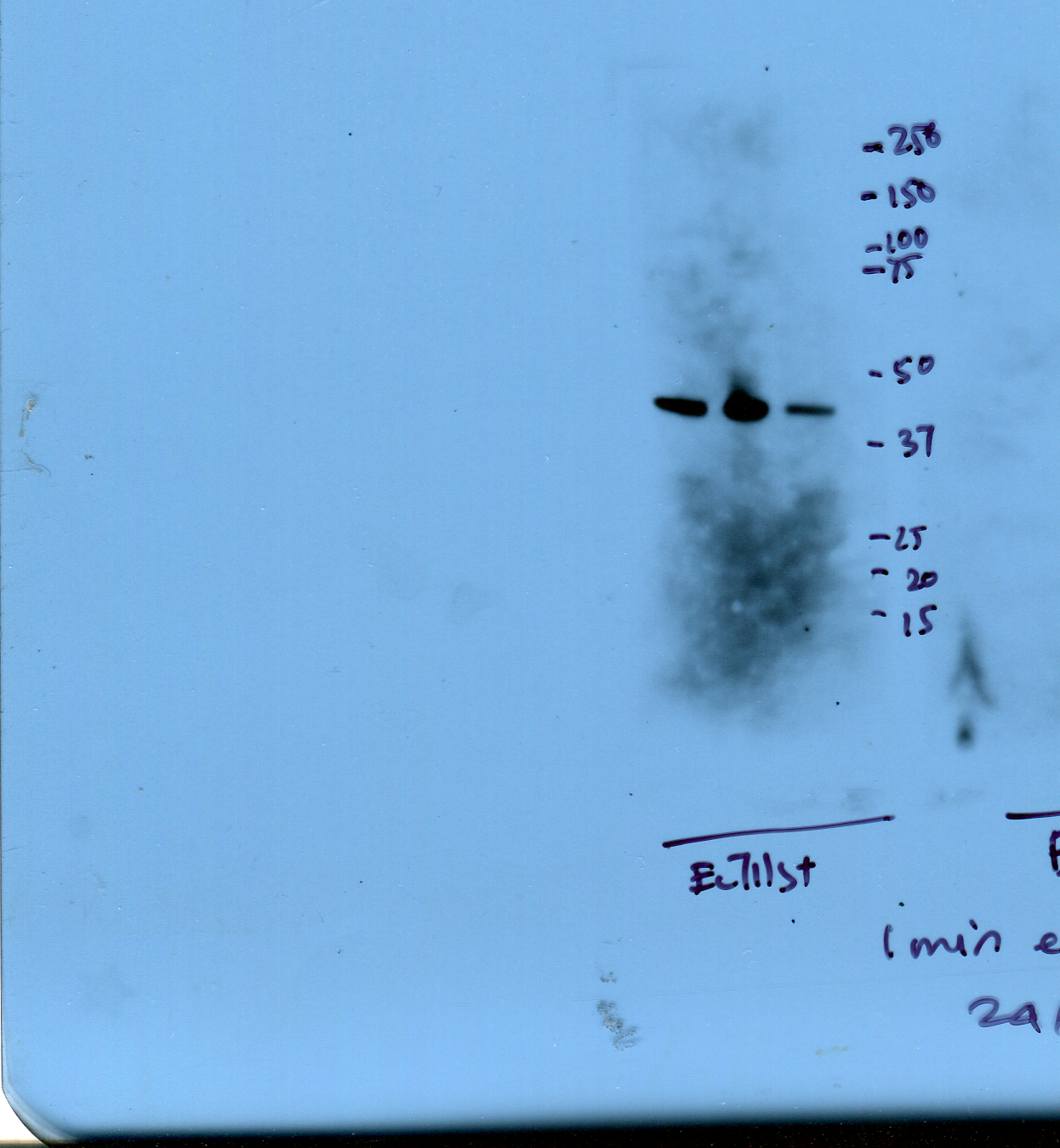

Supplement: Supplementary file 5 — Source data Fig. 4 [file 44319_2025_420_MOESM5_ESM.zip › Figure 4/4D/Fig 4D_Immunoblot_anti-aldolase.tif]

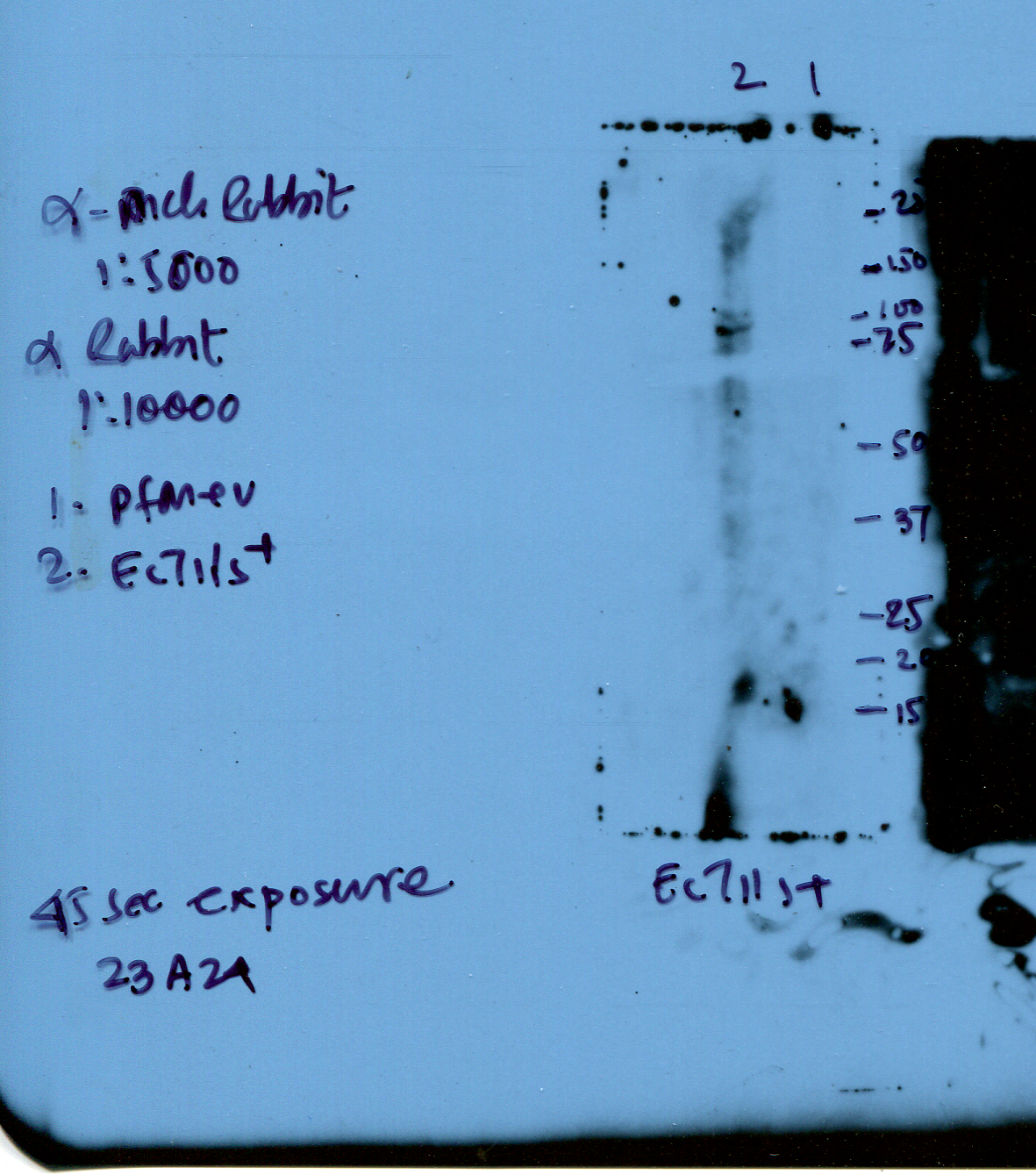

Supplement: Supplementary file 5 — Source data Fig. 4 [file 44319_2025_420_MOESM5_ESM.zip › Figure 4/4D/Fig 4D_Immunoblot_anti-mCherry.tif]

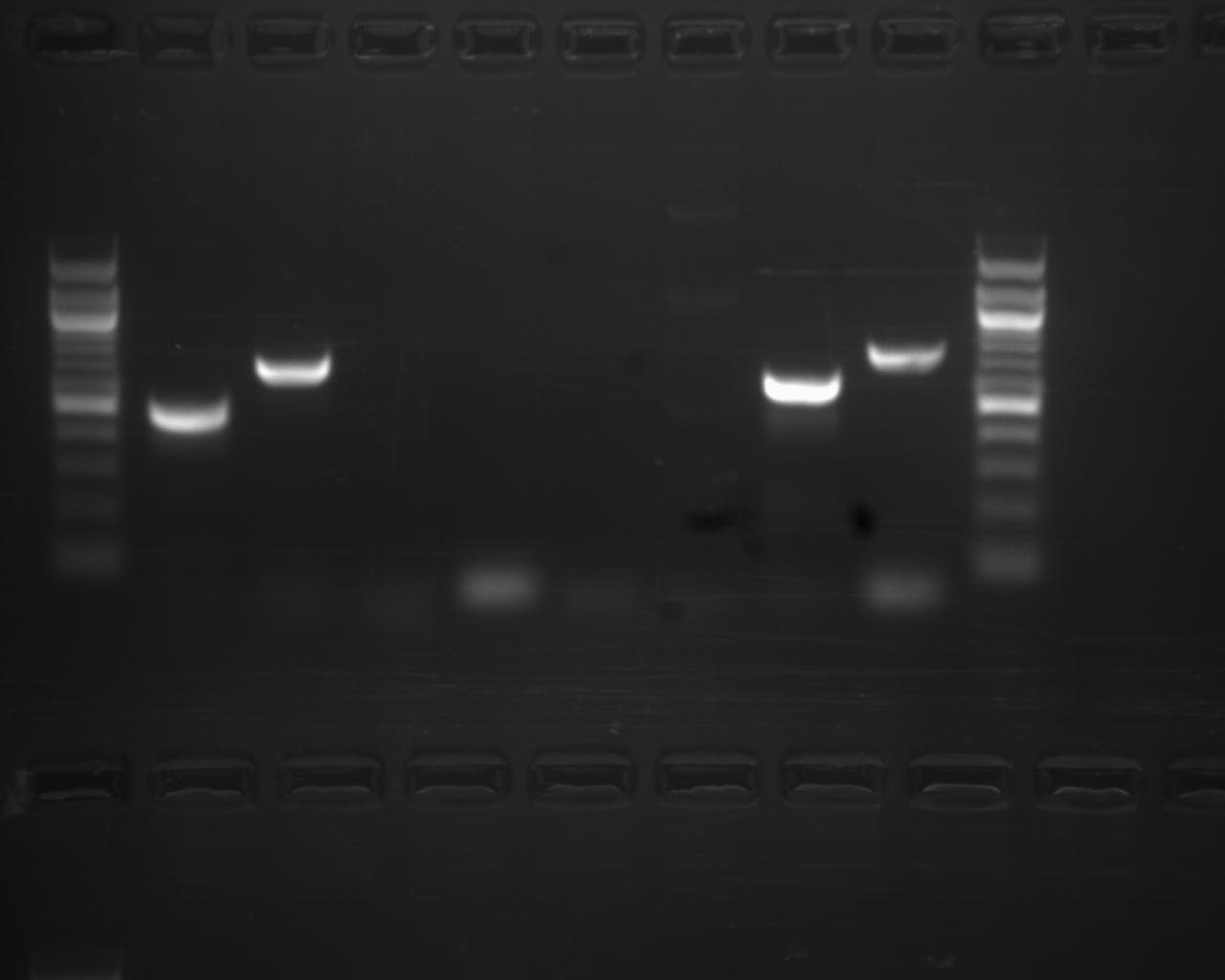

Supplement: Supplementary file 6 — Source data Fig. 5 [file 44319_2025_420_MOESM6_ESM.zip › Figure 5/5B/Fig 5B_genotyping PCR.tif]

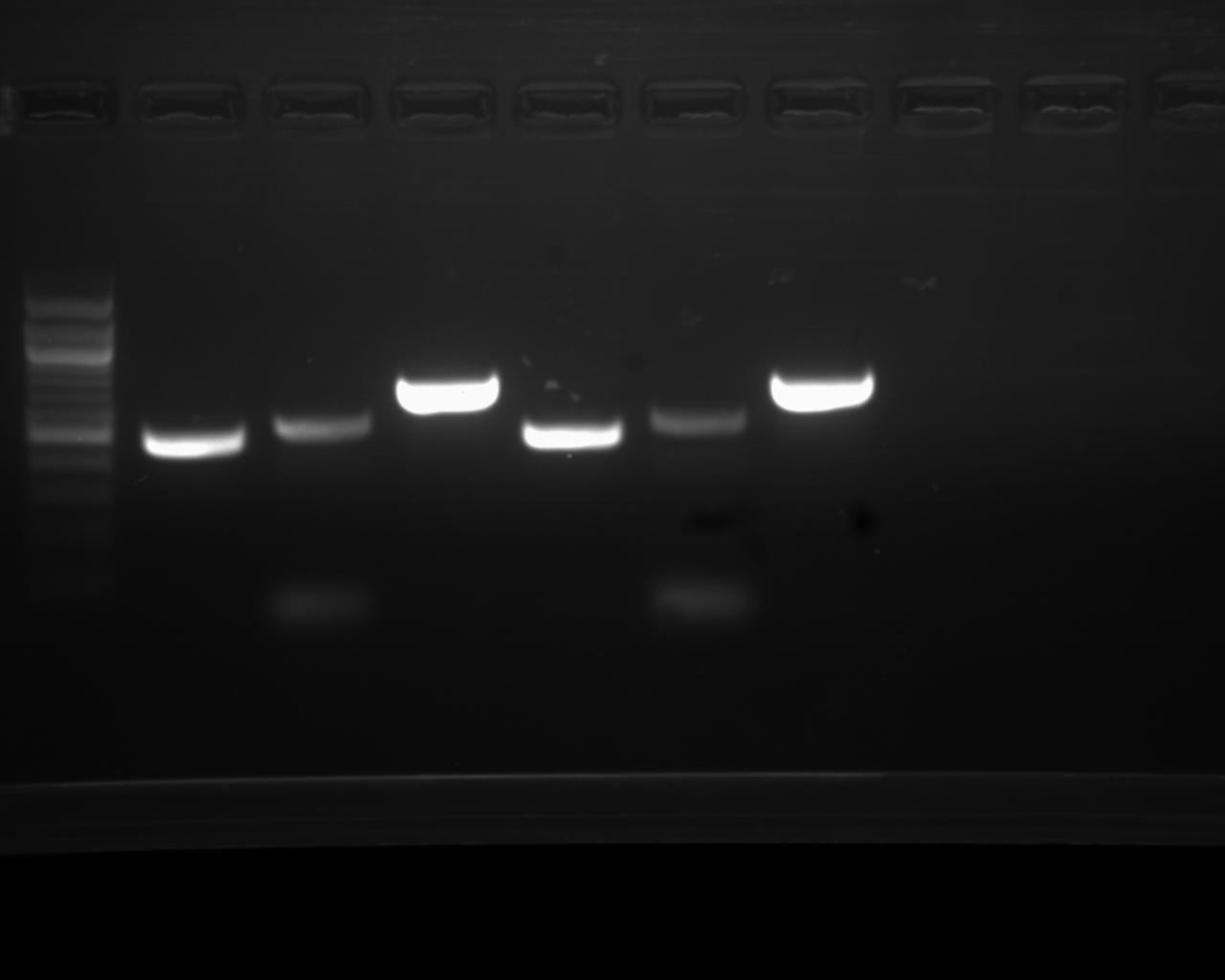

Supplement: Supplementary file 6 — Source data Fig. 5 [file 44319_2025_420_MOESM6_ESM.zip › Figure 5/5E/Fig 5E_NAM PCR.tif]
