# Supplementary material for: tRNA lysidinylation is essential for the minimal translation system in the Plasmodium falciparum apicoplast
Source: EMBO Rep. 2025 Mar 20;26(9):2300–22. doi: 10.1038/s44319-025-00420-w (PMC12069591; doi:10.1038/s44319-025-00420-w)
Supplement: Supplementary file 4 — Source data Fig. 3 [file 44319_2025_420_MOESM4_ESM.zip › Figure 3/3C/Fig 3C readme.pptx]

## Slide 1
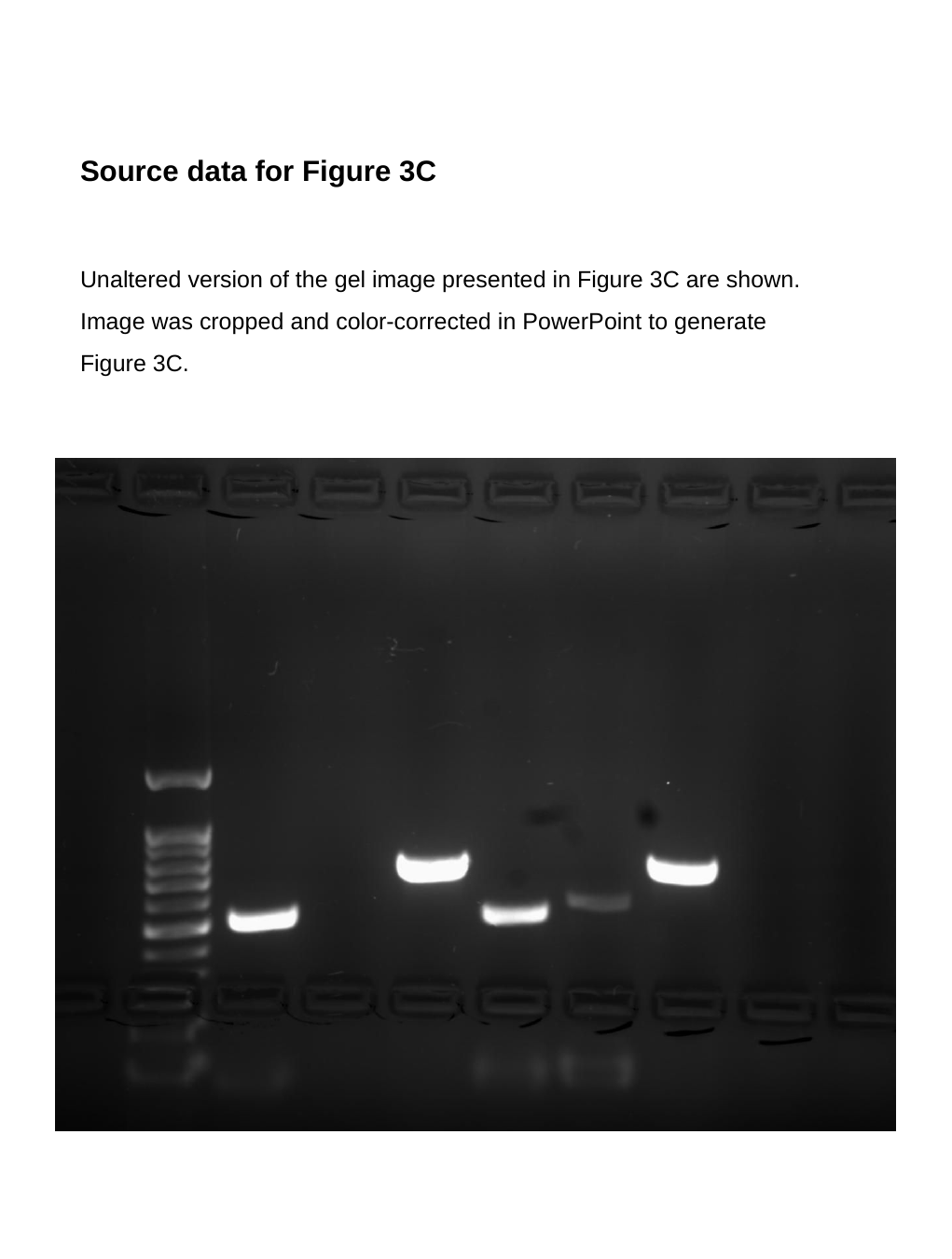

Source data for Figure 3C
Unaltered version of the gel image presented in Figure 3C are shown. Image was cropped and color-corrected in PowerPoint to generate Figure 3C.
