# Supplementary material for: tRNA lysidinylation is essential for the minimal translation system in the Plasmodium falciparum apicoplast
Source: EMBO Rep. 2025 Mar 20;26(9):2300–22. doi: 10.1038/s44319-025-00420-w (PMC12069591; doi:10.1038/s44319-025-00420-w)
Supplement: Supplementary file 5 — Source data Fig. 4 [file 44319_2025_420_MOESM5_ESM.zip › Figure 4/4D/Fig 4D readme.pdf]

Source data for Figure 4D

Unaltered versions of the immunoblots presented in Figure 4D are shown. The red-boxed area was cropped, inverted, and color-corrected using PowerPoint to generate Figure 4D. The uncropped, inverted, and color-corrected versions of full lane immunoblots are provided in Appendix Figure S4.

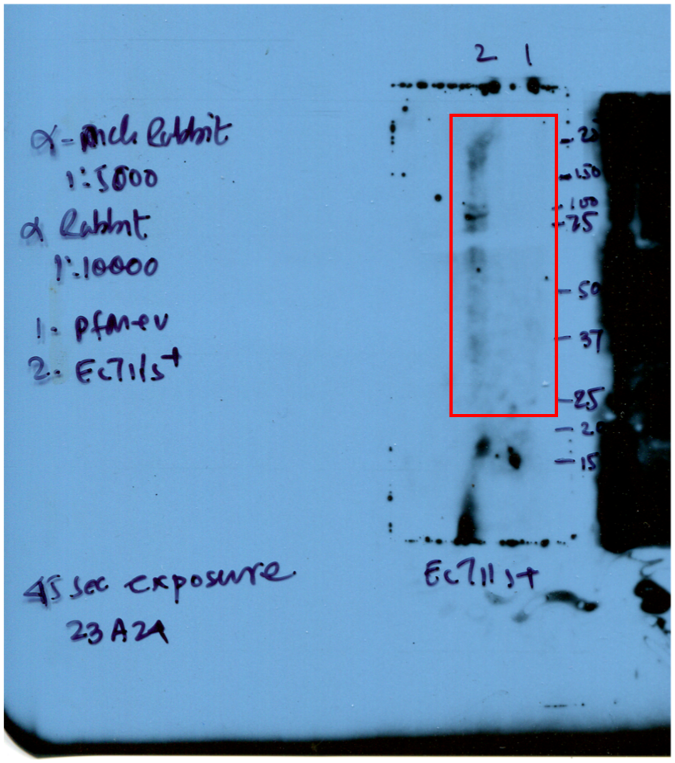

α-mCherry

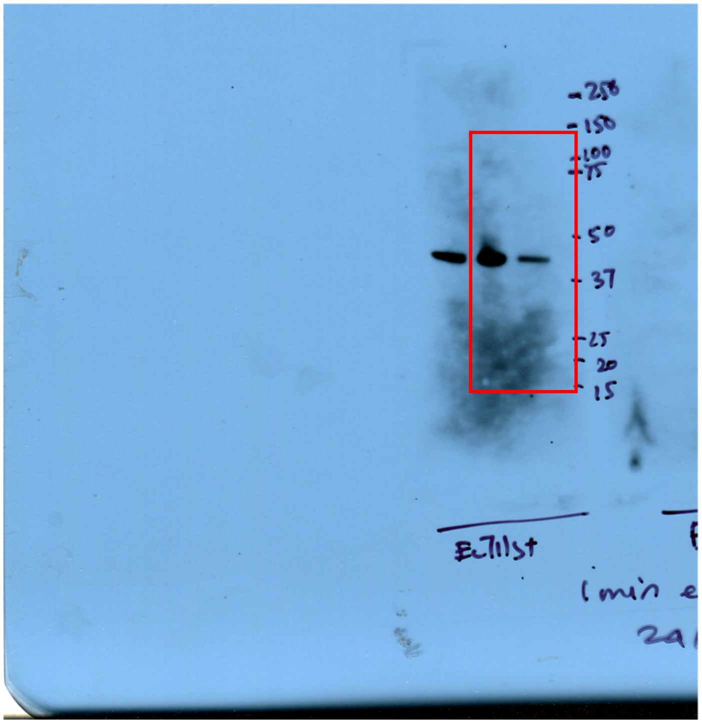

α-aldolase
