# Supplementary material for: tRNA lysidinylation is essential for the minimal translation system in the Plasmodium falciparum apicoplast
Source: EMBO Rep. 2025 Mar 20;26(9):2300–22. doi: 10.1038/s44319-025-00420-w (PMC12069591; doi:10.1038/s44319-025-00420-w)
Supplement: Supplementary file 7 — Expanded View Figures [file 44319_2025_420_MOESM7_ESM.pdf]

Expanded View Figures

aa	Codon	Frequency/ 1000 (Number)	aa	Codon	Frequency/ 1000 (Number)	aa	Codon	Frequency/ 1000 (Number)	aa	Codon	Frequency/ 1000 (Number)
F	UUU	61.7 (458)	S	UCU	17.7 (131)	Y	UAU	104.8 (778)	C	UGU	10.9 (81)
F	UUC	0.7 (5)	S	UCC	0.7 (5)	Y	UAC	1.1 (8)	C	UGC	0.1 (1)
L	UUA	119.6 (888)	S	UCA	13.1 (97)		UAA	3.1 (23)		UGA	0.9 (7)
L	UUG	1.6 (19)	S	UCG	0.4 (3)		UAG	-	W	UGG	3.9 (29)
L	CUU	0.5 (4)	P	CCU	10.2 (76)	H	CAU	9.4 (70)	R	CGU	1.9 (14)
L	CUC	-	P	CCC	0.1 (1)	H	CAC	-	R	CGC	-
L	CUA	1.4 (10)	P	CCA	3.6 (27)	Q	CAA	15.8 (117)	R	CGA	-
L	CUG	-	P	CCG	0.1 (1)	Q	CAG	1.2 (9)	R	CGG	-
I	AUU	70.1 (520)	T	ACU	14.3 (106)	N	AAU	150.5 (1117)	S	AGU	14.6 (108)
I	AUC	1.1 (8)	T	ACC	0.1 (1)	N	AAC	0.9 (7)	S	AGC	-
I	AUA	102.3 (759)	T	ACA	13.6 (101)	K	AAA	123.7 (918)	R	AGA	11.3 (84)
M	AUG	11.6 (86)	T	ACG	0.3 (2)	K	AAG	7.1 (53)	R	AGG	0.4 (3)
V	GUU	6.6 (49)	A	GCU	5.5 (41)	D	GAU	17.3 (128)	G	GGU	16.0 (119)
V	GUC	-	A	GCC	0.1 (1)	D	GAC	0.3 (2)	G	GGC	-
V	GUA	10.6 (79)	A	GCA	2.2 (16)	E	GAA	19.5 (145)	G	GGA	11.5 (85)
V	GUG	0.1 (1)	A	GCG	-	E	GAG	1.1 (8)	G	GGG	1.8 (13)

Figure EV1. Codon usage by 30 proteins encoded in the *P. falciparum* apicoplast genome.

The table presents codon usage frequencies for all proteins encoded by the *P. falciparum* apicoplast genome. Frequencies are expressed as the number of occurrences of each codon per thousand codons, with the total number of occurrences provided in parentheses. Stop codons are shaded in gray. AUA (highlighted in yellow) is the most frequently used codon for isoleucine (I) in apicoplast-genome-encoded proteins. Single-letter amino acid (aa) codes are used. A dash (-) indicates codons that are not used in apicoplast-genome-encoded proteins.

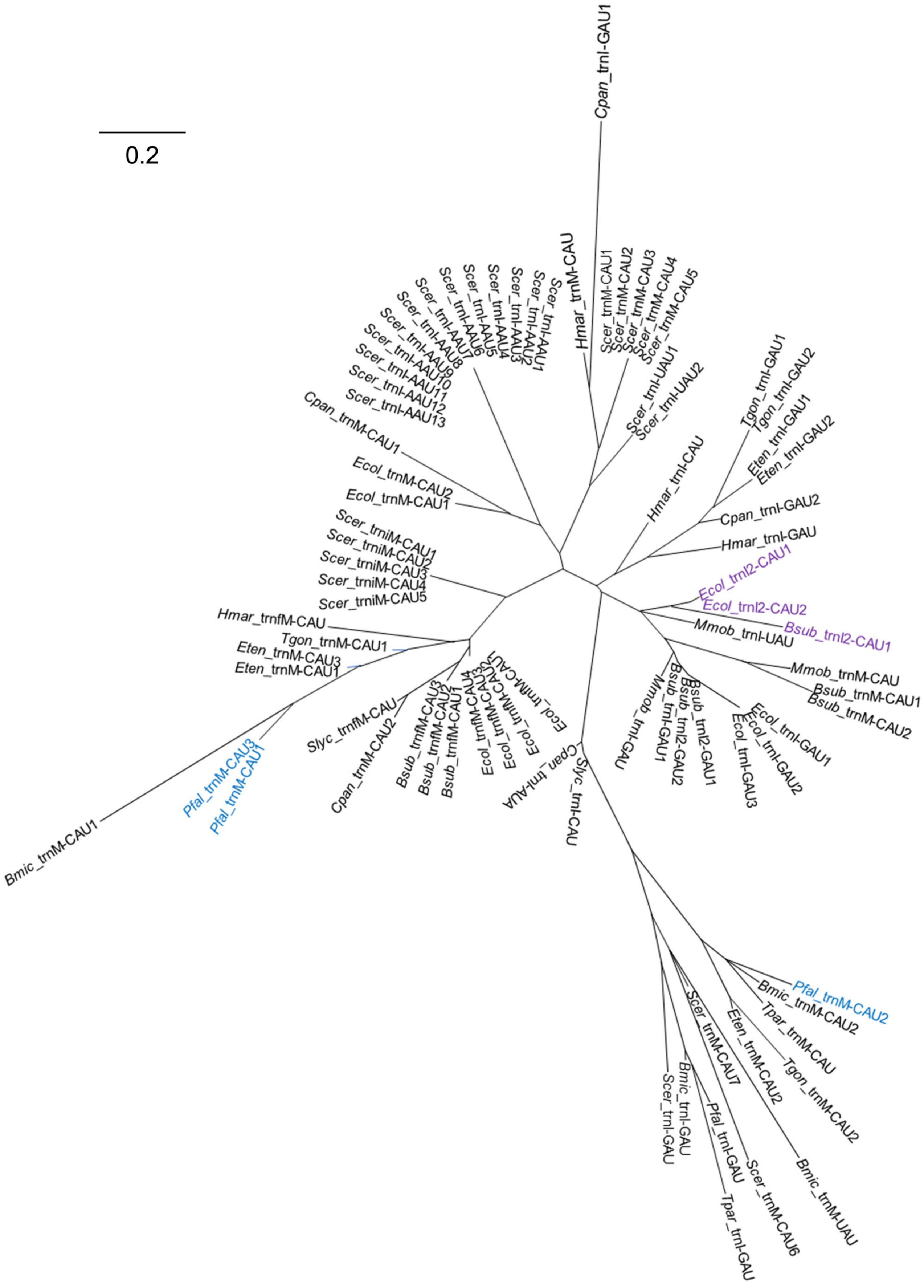
0.2
Cpan_trnI-GAU1
Hmar_trnM-CAU
Scer_trnM-CAU1
Scer_trnM-CAU2
Scer_trnM-CAU3
Scer_trnM-CAU4
Scer_trnM-CAU5
Scer_trnI-UAU1
Scer_trnI-UAU2
Scer_trnI-AAU1
Scer_trnI-AAU2
Scer_trnI-AAU3
Scer_trnI-AAU4
Scer_trnI-AAU5
Scer_trnI-AAU6
Scer_trnI-AAU7
Scer_trnI-AAU8
Scer_trnI-AAU9
Scer_trnI-AAU10
Scer_trnI-AAU11
Scer_trnI-AAU12
Scer_trnI-AAU13
Cpan_trnM-CAU1
Ecol_trnM-CAU2
Ecol_trnM-CAU1
Scer_trniM-CAU1
Scer_trniM-CAU2
Scer_trniM-CAU3
Scer_trniM-CAU4
Scer_trniM-CAU5
Hmar_trnfM-CAU
Tgon_trnM-CAU1
Eten_trnM-CAU3
Eten_trnM-CAU1
Pfal_trnM-CAU3
Pfal_trnM-CAU1
Bmic_trnM-CAU1
Slyc_trnfM-CAU
Cpan_trnM-CAU2
Bsub_trnfM-CAU3
Bsub_trnfM-CAU2
Bsub_trnfM-CAU1
Ecol_trnfM-CAU4
Ecol_trnfM-CAU3
Ecol_trnfM-CAU2
Ecol_trnM-CAU5
Cpan_trnI-AUA
Slyc_trnI-CAU
Tgon_trnI-GAU1
Tgon_trnI-GAU2
Eten_trnI-GAU1
Eten_trnI-GAU2
Hmar_trnI-CAU
Cpan_trnI-GAU2
Hmar_trnI-GAU
Ecol_trnI2-CAU1
Ecol_trnI2-CAU2
Bsub_trnI2-CAU1
Mmob_trnI-UAU
Mmob_trnM-CAU
Bsub_trnM-CAU1
Bsub_trnM-CAU2
Mmob_trnI-GAU
Bsub_trnI-GAU1
Bsub_trnI2-GAU2
Bsub_trnI2-GAU1
Ecol_trnI-GAU1
Ecol_trnI-GAU2
Ecol_trnI-GAU3
Pfal_trnM-CAU2
Bmic_trnM-CAU2
Tpar_trnM-CAU
Tgon_trnM-CAU2
Eten_trnM-CAU2
Scer_trnM-CAU7
Bmic_trnM-UAU
Scer_trnM-CAU6
Pfal_trnI-GAU
Tpar_trnI-GAU
Bmic_trnI-GAU
Scer_trnI-GAU

◀ **Figure EV2. The phylogenetic relationship among methionine- and isoleucine-decoding tRNAs.**

Apicoplast encoded $tRNA_{CAU}$ are shown in blue font with PF3D7_API00600 (*pfal*_trnM-CAU2) sharing common ancestry with experimentally validated TilS substrate $tRNA^{Ile}_{CAU}$ from other species (in purple font). Bootstrap analyses (1000 replicates) were employed to assess the robustness of the branching patterns. The phylogenetic tree is drawn to scale, with branch lengths representing the estimated number of substitutions per site. Refer to Appendix Table S3 for tRNA sequences.

© The Author(s)

A

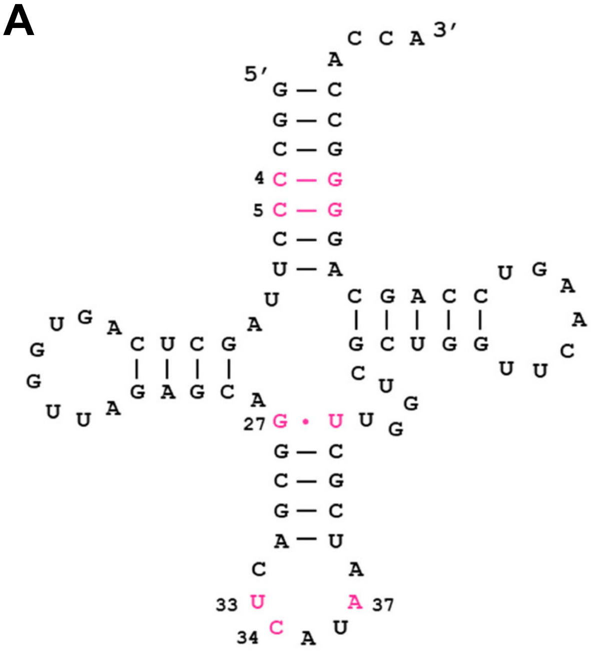

E. coli tRNA$_{CAU}^{Ile}$

B

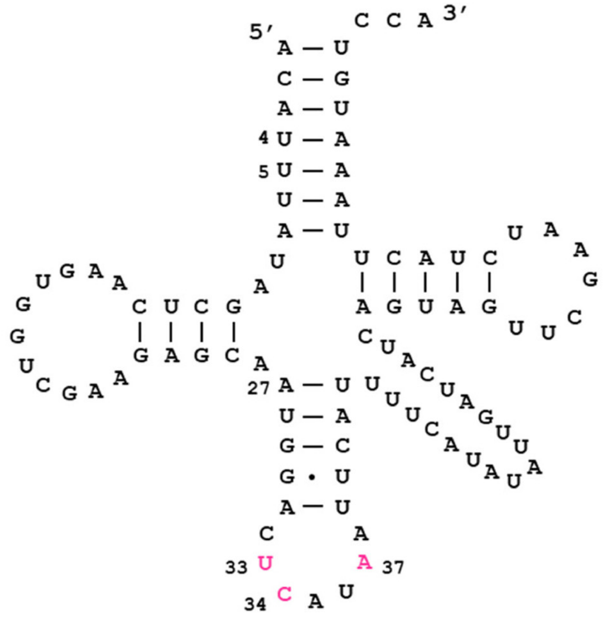

P. falciparum tRNA$_{CAU}$2
PF3D7_API00600

C

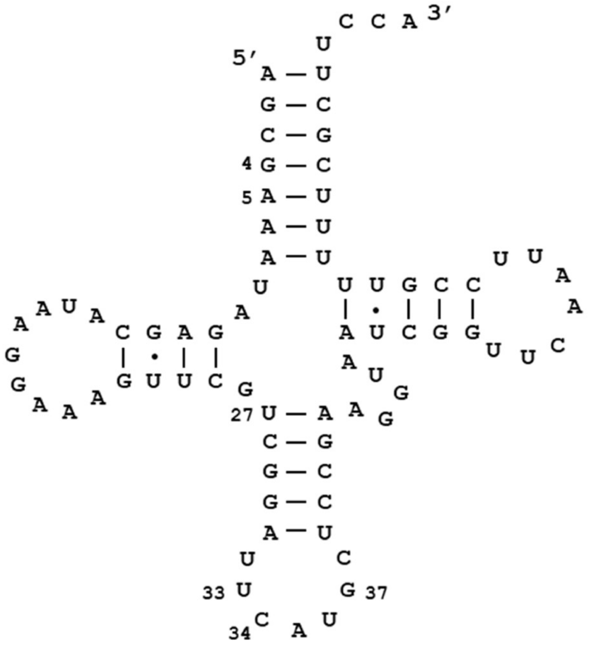

P. falciparum tRNA$_{CAU}$1
PF3D7_API06600

P. falciparum tRNA$_{CAU}$3
PF3D7_API05000

 © The Author(s)

◀ **Figure EV3. Comparison of secondary structures between *Escherichia coli* tRNA$^{Ile}_{CAU}$ and *Plasmodium falciparum* tRNAs from the apicoplast genome which are currently annotated as tRNA$^{Met}_{CAU}$.**

(**A**) *E. coli* tRNA$^{Ile}_{CAU}$. (**B**) PF3D7_API00600. (**C**) PF3D7_API06600 and PF3D7_API05000 (identical sequences due to gene duplication). In (**A**) and (**B**), the positive determinants for TilS binding are shown in magenta. In all panels, the dash (–) indicates canonical base pairing and the dot (.) indicates GU or UU base pairing.
